# The Prevalence of Nutritional Anaemia in Brazilian Pregnant Women: A Systematic Review and Meta-Analysis

**DOI:** 10.3390/ijerph20021519

**Published:** 2023-01-13

**Authors:** Amanda Biete, Vivian S. S. Gonçalves, Sylvia C. C. Franceschini, Eduardo A. F. Nilson, Nathalia Pizato

**Affiliations:** 1 Graduate Program in Human Nutrition, Department of Nutrition, University of Brasilia, Brasilia 70910-900, Brazil; 2 Graduate Program in Public Health, Department of Nutrition, University of Brasilia, Brasilia 70910-900, Brazil; 3 Graduate Program in Nutrition Sciences, Department of Nutrition and Health, Federal University of Viçosa, Viçosa 36570-900, Brazil; 4 Centre for Epidemiological Research in Nutrition and Health (NUPENS), University of Sao Paulo, Sao Paulo 05508-060, Brazil; 5 Oswaldo Cruz Foundation (Fiocruz) Brasilia, Brasilia 70904-130, Brazil

**Keywords:** pregnancy, anaemia, iron, prevalence, Brazil, meta-analysis

## Abstract

Despite the global tendency of maternal anaemia to decline, the persistence of anaemia in Brazil is an important health problem given its vulnerability to deficiencies and the significant increase in nutritional requirements during pregnancy. The aim of this study was to estimate the prevalence of anaemia in Brazilian pregnant women through a systematic literature review and meta-analysis. The systematic review was carried out according to Systematic Reviews and Meta-Analyses PRISMA checklist recommendations and using the following electronic databases: Medline, Scopus, Embase, Web of Science, Lilacs, Scielo, Google Scholar, and CAPES Catalog of Theses and Dissertations. Studies that presented a prevalence of anaemia data in Brazilian pregnant women, considering all gestational trimesters, were included. The total sample included 12,792 pregnant women covering all gestational trimesters. The pooled prevalence of anaemia in Brazilian pregnant women was 23% (95% CI: 20–27), with the highest prevalence in the Northeast Region at 26% (95% CI 23–29), while the lowest prevalence was observed in the North Region with 17% (95% CI 14–20). Among the subgroups, no statistical difference was observed. The prevalence of anaemia status in Brazil is still classified as a moderate public health problem according to the World Health Organization maternal anaemia classification.

## 1. Introduction

Maternal anaemia is one of the most common nutritional deficiencies during pregnancy and is defined as a haemoglobin level below 11 g/dL, according to the World Health Organization (WHO) criteria [1,2]. Maternal anaemia can occur due to several factors, including acute infections, chronic inflammation, and single or combined deficiencies of nutrients such as folic acid, vitamin B12, and iron deficiency, the latter being the most common [3,4,5]. Iron deficiency anaemia occurs due to insufficient iron serum levels in the blood, which reduces the amount of haemoglobin in red blood cells, responsible for transporting oxygen to the body tissues [6]. During pregnancy, this reduction is justified by a hemodilution process, the disproportionate increase in plasma volume to the erythrocyte mass [1]. Further, the reduced woman’s iron deposit is related to the higher serum haemoglobin levels and the developing fetus’ increased demand [5]. 

Iron deficiency anaemia is considered a major contributor to maternal and fetal morbidity and mortality, especially in low and middle-income countries. This anaemia can have serious consequences, such as an increased risk of premature birth, low birth weight, and infant death [1,7], and is considered a worldwide public health problem [8]. In addition, maternal iron deficiency anaemia can affect iron concentrations in the umbilical cord blood of newborns [3], and fetal-neonatal iron deficiency can cause a decrease in auditory recognition memory in infants [9]. Folic acid deficiency is linked with increased neural tube defects, and low maternal erythrocyte folate is also associated with low birth weight and increased risk for small-for-gestational-age (SGA) babies [10,11]. Vitamin B12 deficiency anaemia can affect fetal growth and development, and this deficiency during childhood is associated with neurological complications [12]. Regarding the mothers, symptoms of anaemia include pallor, fatigue, and tachycardia, and depleted blood reserves during childbirth can increase the need for blood transfusions and lead to placental abruption, heart failure, preeclampsia, and death [11]. 

To reduce the prevalence of anaemia in Brazil, two important public policies for the control of iron deficiency were established. In 2004 the fortification of wheat and corn flour with iron and folic acid was mandatory for industrialized foods and from 2005 the supplementation advice of 40 mg of elemental iron for pregnant women, every day, until the end of pregnancy was provided [13].

Although the WHO carries out worldwide periodic monitoring of anaemia in various groups, the last official data regarding the prevalence of anaemia in pregnant women in Brazil was published by the WHO in 2015. This data estimated that the global prevalence of anaemia for pregnant women aged 15–49 years in 2011 was 38.2% (95% CI 33–42), the African region presented 46.3 (95% CI 40–51), region of the Americas 24.9% (95% 19–32), European region 25.8% (95% CI 19–33) and South-East Asia region 48.7% (95% CI 36–58). Brazilian pregnant women presented a prevalence of 32% (95% CI 11–62) [4]. The global prevalence of anaemia in pregnant women decreased from 43% (95% CI 39–47) to 36% (95% CI 34–39) considering the period 1995 to 2019 [14,15], but it is still considered a moderate-to-severe public health problem [4]. Regarding Brazilian pregnancy rates, the WHO Global Health Observatory reports in 2019 the estimated prevalence of 19.1% (95% CI 9.7–38.0), which was classified as mild-to-moderate prevalence [16].

Maternal anaemia in Brazil has been studied since the 1970s, especially in the period between 1970 and 1990. After this period, there was a reduction in the number of studies investigating the prevalence of anaemia in pregnant women [17]. Brazil is facing a nutritional transition characterized by the existence of a double load of diseases, contemplating the steady rise in obesity prevalence associated with the persistence of anaemia in the maternal–infant group [18]. Considering the anaemia as a nutritional deficiency, the absence of recently published national data, and the scarcity of nationally representative studies, we conducted a systematic review and meta-analysis to update the prevalence of anaemia in pregnant women in Brazil. 

## 2. Materials and Methods

This systematic review was conducted according to the Preferred Reporting Items for Systematic Reviews and Meta-Analyses PRISMA checklist [19] (Table S3) and registered in the International Prospective Register of Systematic Reviews (PROSPERO), number CRD42021261303. 

### 2.1. Eligibility Criteria

The eligibility criteria were studies that presented anaemia prevalence data in healthy pregnant women in Brazil, considering all gestational trimesters, and presented defined criteria for anaemia diagnosis. Subjects from 10 to 19 years of age were considered adolescents and women over 19 years old were considered adults [20]. 

The following criteria were excluded from the review: studies conducted with unhealthy pregnant women, self-reported anaemia, letters to the editors, reviews, personal opinions, book chapters, comments, editorials, and any publication without primary data. 

The searches were carried out by two authors with systematic review expertise in English and Portuguese languages and no restrictions regarding the publication date. 

### 2.2. Information Sources

The search strategy was performed according to the criteria established by the Peer Review of Electronic Search Strategies (PRESS checklist) [21]. The article search was carried out on 29 June 2021 and updated on 10 November 2022. The electronic databases Medline, Embase, Scopus, Web of Science, Lilacs, and Scielo were used. Gray literature studies that met the eligibility criteria established in this review were examined using Google Scholar searching in English and Portuguese. Brazilian thesis and dissertation research were identified by searching abstracts in the CAPES thesis and dissertation directory. Reference lists of entered articles were manually searched to identify studies not retrieved in the databases.

Missing data were sought via emails to the corresponding authors. Records were downloaded to a Microsoft Excel spreadsheet, added to a standardized data collection form, and duplicates were removed using Mendeley^®^ software. Rayyan^®^ software was used for checking possible duplicate references and to triage potentially eligible studies.

### 2.3. Search Strategy

The keywords used in the search strategy were found in Medical Subject Headings and Health Sciences Descriptors. The search strategy was reviewed by researchers experienced in performing systematic reviews, according to the checklist of the Peer Review of Electronic Search Strategies (PRESS checklist) [21].

The search strategy was adapted for each database using the following Boolean descriptors and operators: (Pregnancy OR “Pregnant women” OR Gravidity OR Pregnant OR Gravid OR Antenatal OR Antepartum OR Gestation) AND (Brazil OR Brazilian) AND (anemia OR anaemia OR haemoglobin OR hemoglobin OR haematocrit OR hematocrit OR “iron deficiency anemia” OR “iron deficiency anaemia” OR “human hemoglobin” OR “human haemoglobin” OR “hemoglobin levels” OR “haemoglobin levels” OR “Iron deficiency” OR “Iron-Deficiency Anemia” OR “Maternal anemia” OR ferritin) AND (“Observational Study” OR “Cohort study” OR “Longitudinal study” OR “Follow-up study” OR Cohort OR Longitudinal OR Prospective OR Retrospective OR “Incidence study” OR Follow-up OR “Prevalence study” OR Prevalence OR “Cross-Sectional Study” OR Cross-Sectional OR frequency).

The search performed on Google Scholar was limited to the first 200 most relevant articles [22]. None of the language, publication date, or status filters were applied to the results of each database. Search strategies are detailed in Table S1.

### 2.4. Studies Selection

The study selection process was carried out in two stages. Firstly, the titles and abstracts of the retrieved articles were screened. Then, the remaining citations were fully read to evaluate the potentially eligible studies. Studies were included if the eligibility criteria were met. Then, data were extracted from the studies that remained. In cases of divergence between authors, the decision to include or exclude an article was taken by consensus.

### 2.5. Data Extraction

Data were extracted by two independent authors and entered into an Excel spreadsheet containing the following fields: author’s name, year of study, year of publication, Brazil geographic region, study design, local data collection, age of pregnant women, gestational trimester, sample size, method of haemoglobin and haematocrit collection, the prevalence of anaemia and uses of antianemics supplementation. At least two attempts were made to contact authors to obtain additional information or to request the full text when it was not available in the articles. 

Some studies did not provide 95% CI for the prevalence of anaemia, so it was calculated through the website Open Epi [23], using the sample size and the number of events in the sample. 

### 2.6. Appraisal of Methodological Quality

The JBI Critical Appraisal Checklist for Studies Reporting Prevalence Data by the Joanna Briggs Institute was used to assess the methodological quality of the studies [24]. 

The instrument consists of nine questions and four answers (yes, no, unclear, and not applicable): (1) Was the sample frame appropriate to address the target population? (2) Were study participants recruited in an appropriate way? (3) Was the sample size adequate? (4) Were the study subjects and setting described in detail? (5) Was data analysis conducted with sufficient coverage of the identified sample? (6) Were valid methods used for the identification of the condition? (7) Was the condition measured in a standard, reliable way for all participants? (8) Was there appropriate statistical analysis? (9) Was the response rate adequate, and if not, was the low response rate managed appropriately? [24]. 

The risk of bias was considered low when the answer to all items was “yes” and high if the answer was “no” or “unclear” to at least one of the items. The risk of bias assessment was used as a parameter in the heterogeneity analysis of the included studies; however, it was not used as a criterion for their exclusion.

### 2.7. Summary Measures and Data Analysis

The primary outcome of the analysis was the anaemia prevalence with a 95% confidence interval (95% CI). In addition, the qualitative analysis included articles considering gestational trimesters, age, geographic region, use of antianaemic supplementation, studies before and after fortification and supplementation public policies, and study publication period (before and after 2011).

The meta-analysis was performed using random effects models and the inverse of variance method. The magnitude of heterogeneity of the studies was verified by the Higgins and Thompson I-square (I²) [25]. A forest plot was constructed of the prevalence and confidence intervals of each study.

### 2.8. Assessment of Heterogeneity and Publication Bias

Subgroups analysis and meta-regressions were performed to verify the source of heterogeneity in the studies included in the systematic review. In the subgroup analysis, the following covariates were used: sample size (≤500 and >500), age (adolescent, adult and adolescent, and not reported), Brazil geographic region (North, Northeast, South, Southeast, and Midwest), period of publication (before or after 2004 and 2011), measured gestational trimester (all, only in one or two trimesters, and not reported), Hb determination method (capillary or venous) and methodological quality (high or low). 

Publication bias was assessed using the funnel plot and the calculation of the Egger test [26], with a significance of *p* < 0.05. Data analysis was performed using STATA^®^ version 16.

### 2.9. Quality of Meta-Evidence

The Grading of Recommendations Assessment, Development, and Evaluation (GRADE system) was used to summarize the overall quality of the evidence from pooled studies. The evidence score started at high-quality evidence and was downgraded by one or two levels if one of the following pre-specified criteria was present: (1) Risk of Bias (considering inappropriate sampling method or statistical analyses in more than 75% of studies); (2) Inconsistency (heterogeneity was considered important when I^2^ presents values of more than 40%); (3) Indirectness (downgrade if less than 25% of studies did not use valid and reliable methods for data collection); (4) Imprecision (downgrade for imprecision if more than 75% of studies had a small sample size (≤500); (5) Publication bias (was considered when the significance of *p* < 0.05). 

## 3. Results

### 3.1. Selected Studies

**Figure 1:**
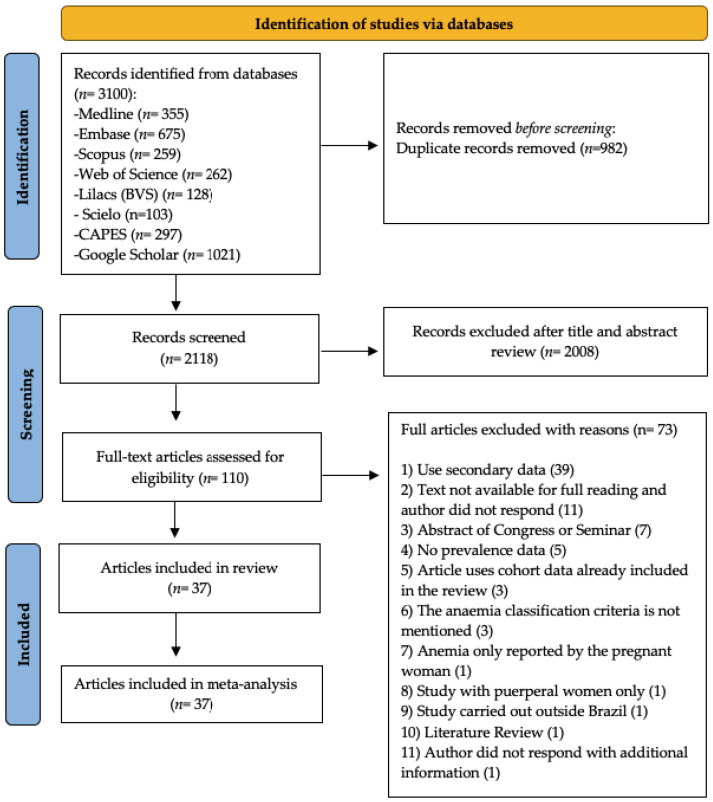
Flowchart of the study selection process. Adapted from PRISMA.

The search in eight electronic databases, including the gray literature and Google Scholar, resulted in 3100 records identified. After removing duplicates, a total of 2118 was screened based on titles and abstracts. One hundred and ten articles with the potential for full text were read. Of these, 73 were excluded due to not meeting eligibility criteria (Figure 1). Table S4 shows the reasons for excluding each article. After a complete reading, 37 [27,28,29,30,31,32,33,34,35,36,37,38,39,40,41,42,43,44,45,46,47,48,49,50,51,52,53,54,55,56,57,58,59,60,61,62,63] articles were included in this systematic review.

### 3.2. Characteristics of the Studies

The total sample included 12,792 pregnant women covering all gestational trimesters. The gestational week varied between seven [35] and 42 [40] and the age between 10 [29] and 49 years [29,58]. Thirty four studies (91.89%) presented cross-sectional design [27,28,29,30,31,32,33,34,35,37,38,39,40,41,42,43,44,45,46,47,48,49,50,51,52,53,54,56,57,58,59,60,61,62] and three (8.11%) were cohorts [36,55,63]. Among the selected articles, two studies were carried out with only pregnant adults [34,58] and four studies evaluated only adolescents [30,41,49,61]. The years of publication ranged from 1974 [57] to 2021 [56]. Of the articles included, 26 (70.27%) [27,29,33,34,36,37,38,42,45,46,47,48,49,50,52,53,54,55,56,58,59,60,62,63] were carried out after 2004, the year of implementation of the public policy for fortification of wheat and corn flour with iron and folic acid, and 12 (32.43%) [27,35,36,38,46,48,50,52,53,54,55,63] were carried out after 2011, the year of the last anaemia prevalence data published by the WHO in 2015 [4]. 

The majority of the studies were carried out in the Northeast region (*n* = 16; 43.24%) [27,28,34,38,39,42,46,47,50,51,52,53,54,56,62], followed by the Southeast region (*n* = 11; 29.73%) [30,31,32,40,41,44,49,55,57,61]. Four studies evaluated the Midwest region (10.81%) [33,45,58,59], two the North region (5.41%) [48,60], and four the South region (10.81%) [29,35,36,37]. Table 1 summarizes the main results of the studies.

### 3.3. Methodological Quality of Individual Studies

**Figure 2:**
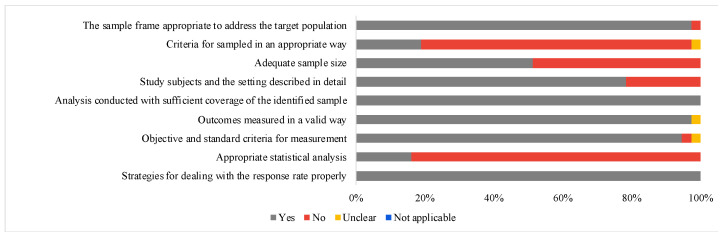
Risk of bias of the included articles according to study design.

The 37 studies evaluated were considered heterogeneous in terms of methodological quality. Only two studies [38,59] were considered low risk of bias; the other 35 were considered highly biased since they presented one or more items classified as “no” or “unclear”. Among the nine parameters evaluated, the “Analysis conducted with sufficient coverage of the identified sample” and “Strategies for dealing with the response rate properly” were evaluated with “yes” in 100% of the articles. On the other hand, the parameters “Appropriate statistical analysis” and “Criteria for sampled in an appropriate way” were the least attended, with a “no” answer for 83.78% and 78.37%, respectively. The risk of bias analysis is shown in Figure 2 and Table S2. 

### 3.4. Results of Individual Studies

Thirty-five studies [27,28,29,30,31,33,34,35,36,37,38,39,40,41,42,43,44,45,46,47,48,49,50,51,52,53,54,55,56,58,59,60,61,62,63] used the criteria proposed by the WHO for the diagnosis of anaemia (1972), which defines anaemia as a haemoglobin concentration <11 g/dL. The other criteria used were Hb < 11.6 g/dL [32] and Hb < 12 g/dL [57]. Regarding the method used for identifying blood haemoglobin rates, the majority (*n*=31; 83.78%) [28,29,30,31,32,35,37,39,40,41,42,44,45,46,47,48,49,51,53,54,55,56,57,58,60,61] used venipuncture. The data collected came from public health services: community (*n* = 3; 8.11%) [36,50,62], hospitals (*n* = 4; 10.81%) [27,40,44,52], maternity hospitals (*n* = 4; 10.81%) [28,30,39,51], primary health units (*n* = 17; 45.95%) [29,31,32,34,37,38,42,43,46,47,48,53,54,56,60,61,63], prenatal clinic (*n* = 2; 5.41%) [49,55], philanthropic unit (*n* = 1; 2.70%) [57], universities (*n* = 1; 2.70%) [41], and university hospitals (*n* = 5; 13.51%) [33,35,45,58,59].

The prevalence of anaemia reported in the studies ranged from five [58] in the city of Cuiabá (Midwest region) to 52.3% [57] in the city of São Paulo (Southeast region). 

Only four articles evaluated pregnant adolescents. The age of the participants ranged from 13 [30,49] to 19 [49] years and the prevalence of maternal anaemia ranged from 10 [61] to 41.6% [49]; the latter study was carried out only with adolescents in the third trimester of pregnancy. The four studies were carried out in the Southeast region of Brazil, with three in the state of São Paulo [30,41,61] and one in the state of Rio de Janeiro [49].

Regarding antianaemic supplementation, 24 studies [27,29,30,33,34,36,38,39,42,43,44,46,47,48,49,50,51,52,53,54,59,61,62,63] published between 1991 [44] to 2019 [46,63] presented data about supplementation. Of these studies, 4884 (55.06%) used antianaemic supplementation and 14 studies [29,36,38,39,42,44,46,47,49,51,52,53,54,63] analyzed anaemia in supplemented and non-supplemented pregnant women. The prevalence of anaemia in supplemented pregnant women ranged from 1.61 [53] to 60.67% [36], and in the non-supplemented group ranged from 7.5 [42] to 100% [49]. Among the studies performed with adolescents, two analyzed antianaemic supplementation, and the prevalence of supplemented pregnant adolescents was 34.1% [30] and 54% [61].

### 3.5. Meta-Analysis

**Figure 3:**
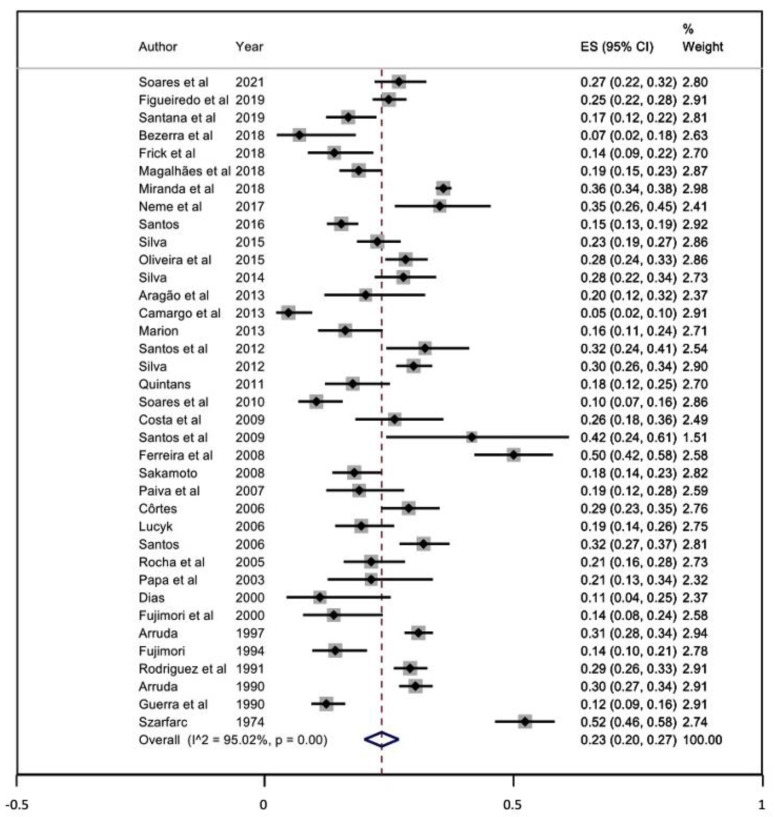
Prevalence of anaemia in Brazilians pregnant women [27,28,29,30,31,32,33,34,35,36,37,38,39,40,41,42,43,44,45,46,47,48,49,50,51,52,53,54,55,56,57,58,59,60,61,62,63].

To estimate the national prevalence of anaemia in pregnant women, 37 [27,28,29,30,31,32,33,34,35,36,37,38,39,40,41,42,43,44,45,46,47,48,49,50,51,52,53,54,55,56,57,58,59,60,61,62,63] studies were included in the meta-analysis. The prevalence of anaemia in the Brazilian pregnant women group was 23% (95% CI 20–27; I^2^ = 95.02), as shown in Figure 3, and in the adolescent group 19% (95% CI 10–28; I^2^ = 65.62). Considering the fortification policies, before and after 2004, the prevalence of anaemia was 24% (95% CI 17–30) and 23% (95% CI 19–28), respectively (Figure S1a). The included studies published before 2011 present a prevalence of 24% (95% CI 19–28) and after 2011 23% (95% CI 18–28) (Figure S1b). Among the subgroups analysis, no statistical difference was observed (Table 2). When stratified by regions, the Northeast region presented the highest prevalence at 26% (95% CI 23–29), while the lowest prevalence was observed in the North region with 17% (95% CI 14–20) (Figure S1c). No significant difference was observed regarding cicle of life (Figure S1d), risk of bias (Figure S1e) and Hb determination method (Figure S1f).

According to the funnel graph shown in Figure S2, no publication bias was shown, confirmed by the Egger test (*p* = 0.090). 

### 3.6. Certainty of Evidence

The overall quality of the evidence was classified as very low quality (⊕◯◯◯), as shown in Table 3. 

## 4. Discussion

We believe that this is the first systematic review conducted with a meta-analysis presenting the prevalence of anaemia in Brazilian pregnant women. The individual studies showed conflicting results regarding the prevalence of anaemia in Brazilian pregnant women, even when the same methods procedures were applied at the same regions. Despite the efforts such as food fortification policies to reduce anaemia prevalence, the result of the meta-analysis showed that anaemia in Brazilian pregnant women was classified as a moderate public health problem (≥20%), regardless of the age group, geographic region, and gestational trimester, according to the WHO maternal anaemia classification [64]. 

The WHO estimated the overall prevalence of global anaemia in pregnant women at 36% (95% CI 34–39) in 2019 [15]. High-income countries have a lower prevalence (15%; 95% CI 10–22), while a higher prevalence is found in West and Central African countries (52%; 95% CI 50–55), followed by South Asian countries (48%; 95% CI 43–52) [15]. Latin America and the Caribbean presented a prevalence of 22% (95% CI 16–29) in 2019 [15]. In Brazil, the WHO published pregnant women anaemia analysis in 2020, which showed an estimated prevalence of 19.1% (95% CI 9.7, 38.0) [16]. 

High rates of anaemia in pregnant women were identified in all the Brazilian regions. The Northeast region has cities with the greatest socio-economic inequality in Brazil and, therefore, a higher prevalence of anaemia was expected. The Midwest and North regions were classified as having mild public health urban problems; however, it is noteworthy that these regions had four and two studies included, respectively, and this result should be interpreted carefully. A literature review study evaluated the last 40-year prevalence of anaemia in Brazilian pregnant women and evidenced that studies were regionally concentrated in the states of São Paulo and Pernambuco until 2009 [17]. The authors also showed that more articles were found investigating anaemia in children’s groups than in women of childbearing age or pregnant women groups. A recent systematic review presented the prevalence of iron deficiency anaemia in Brazilian children under five years of age, but no pregnant women were evaluated [8]. In addition, a review published in 2022 showed the prevalence of 25% (95% CI 23–28), mainly in the North and Northeast regions (30%; 95% CI 24–37), of iron deficiency anaemia in women of childbearing age (a broader target audience than our review), highlighting that anaemia remains a public health problem in Brazil [65].

The prevalence of anaemia in the group of pregnant adolescents was not significantly different from that of adults. However, different outcomes were expected, since adolescents are more likely to be anaemic during pregnancy, due to their higher nutritional requirements and the growth of immature body tissues [66]. The number of studies included in the review and their geographic region may have contributed to these findings, since only four studies with pregnant adolescents were part of the review and all were conducted in the Southeast region. There was also no difference between groups of pregnant women in the first and second trimester of pregnancy, and between all gestational trimesters. Additionally, it was not possible to carry out analysis with only pregnant women in the last gestational trimester group, when greater nutritional requirements are needed and a higher prevalence of anaemia is expected, which may explain the results found. The pregnant adolescent group deserves special attention from health authorities and the encouraging adherence to iron supplementation is considered a very important strategy to mitigate adverse effects of anaemia in this group.

There is recent evidence showing that sampling errors, such as the type of blood sample measurement (venous or capillary), can influence the distribution of haemoglobin in the blood and, consequently, the prevalence of anaemia in studies with similar populations [67]. However, in our study, the meta-analysis showed no difference in the prevalence of anaemia considering the type of blood collection.

The WHO considers iron deficiency anaemia to be the main cause of maternal anaemia and the prevalence of anaemia is used as a proxy indicator to estimate iron deficiency at population level [68]. Maternal anaemia can lead to severe complications for both mother and baby, such as an increased risk of premature birth, low birth weight, and infant and maternal death [1,7]. The Brazilian government instituted two public policies to assist in the control of iron deficiency anaemia in different populations, especially in pregnant women and children. The first is the mandatory fortification of wheat and corn flour with iron and folic acid implemented in June 2004 [69], with provides for the mandatory addition of 4.2 mg of iron and 150 μg of folic acid in wheat and corn flour for every 100 g of the product [8,70,71].The second is the National Iron Supplementation Program implemented in 2005 [8], which recommends supplementation of 40 mg of elemental iron for pregnant women, every day, until the end of pregnancy [13]. Food fortification is suggested by the WHO as the most cost-effective way to reduce the prevalence of anaemia, so, in addition to Brazil, other South American and Central American countries have also implemented food fortification, such as Costa Rica, Chile, El Salvador, Guatemala, Honduras, Mexico, Nicaragua, and Panama, among others [72]. The period of publication of the studies was categorized before or after 2004 to assess the evolution of anaemia prevalence considering the public policies. However, the analysis categorized by the year of study data collection did not show a significant difference as expected. These results may suggest that the implementation of public policies to reduce anaemia may not have been effective for the pregnant women group and, therefore, it is necessary to intensify actions to ensure the effectiveness of intervention strategies. 

On the other hand, only the prevalence of anaemia was evaluated in this review, considering the serum level of haemoglobin. Anaemia may not be caused only by iron deficiency, and in these cases, fortification of flours with iron and folic acid and iron supplementation will have a limited impact on haemoglobin levels and, consequently, on the prevalence of maternal anaemia. This result is corroborated by a systematic review of the effectiveness of flour fortification programs on iron status and anaemia, carried out in 2015 in several countries in the target audience, including the general population, children, and women of reproductive age (including pregnant women). The study concludes that there is limited evidence of the effectiveness of flour fortification in reducing the prevalence of anaemia; the evidence of efficacy in reducing the prevalence of low ferritin in women is more consistent and results may not yet reflect the impact of changes in the flour fortification policies in Brazil, such as not allowing the use of low bioavailable iron. The authors suggest the use of specific biomarkers for the nutrients added to the flour and the non-exclusive use of anaemia biomarkers to assess the impact of flour fortification.

Regarding iron supplementation, the WHO believes that it would increase the mean concentration of serum haemoglobin by 10.2 g/L (95% CI 6.1–14.2) in pregnant women [4]. According to the Organization, when applying these changes in haemoglobin concentrations, about 50% of anaemia in women could be eliminated by iron supplementation. It is known that some factors can contribute to low adherence of pregnant women, such as side effects (gastrointestinal, for example), forgetfulness, difficulty in accessing the supplement, or even weaknesses in the guidelines offered to pregnant women in the public health service [73]. Additionally, the fragility of supplementation program monitoring and changes in the funding of the national iron supplementation program, including the decentralization of supplement purchases to municipalities since 2013, may have influenced the access to iron supplements by pregnant women in Brazil. Thus, it is important to train health professionals to communicate effectively with pregnant women during prenatal care to ensure the elemental iron, ferrous sulfate, and folic acid supplementation adherence as recommended by public policies and to strengthen national program monitoring.

The prevalence of anaemia in Brazilian pregnant women has not changed significantly since the last WHO publication in 2015 with data from 2011. Precarious sociodemographic conditions, such as not having water supply through the general network, food insecurity, low consumption of foods rich in iron, low family income, and low education are considered risk factors for anaemia and hinder the reduction in prevalence [8,29,38,62]. We emphasize the importance of government action with socioeconomic policies to contribute to the reduction of anaemia in the public. Reducing anaemia in women aged 15 to 49 years is a commitment that Brazil made through the United Nations Sustainable Development Goals (SDGs), which proposed to reduce the prevalence of anaemia among women of childbearing age by half by 2030 [15,74]. In addition, Brazil is facing a nutritional transition, which is characterized by a reduction in malnutrition and an increase in obesity in the population, because of the change in the population’s eating habits. The increased food intake of low nutritional quality diet and high energy value, and the reduced consumption of healthy foods, such as fruits and vegetables, contributes to a lower micronutrient intake [75]. Brazil’s nutritional transition has a singularity: the worsening of anaemia [76]. According to an analysis of food consumption in Brazil carried out through the Household Budget Survey, in adolescents aged 10 to 18 years the inadequacy of iron intake increased from 15.1% in 2008–2009 to 20.3% in 2017–2018. Among adult women, inadequacy reached 30.4% in the 2017–2018 survey [77]. Therefore, it is important to reinforce health and dietary policies to promote adequate healthy food intake as the first strategy to prevent anaemia.

According to the GRADE system, the pooling of studies of the prevalence of anaemia in Brazilian pregnant women provided very low-quality evidence. It is worthy to clarify that to estimate the prevalence of anaemia outcomes, it is necessary to assess observational studies in the revision, and the GRADE system considered observational studies at a “low quality” level of evidence. Thus, the GRADE result does not necessarily mean that there is a limitation of the evidence. Regarding the individual assessment of the risk of bias, it is observed that there was no significant difference in the prevalence of anaemia between studies evaluated as low risk of bias and those with a high risk of bias.

Despite the efforts of health authorities to reduce anaemia in pregnant women in Brazil, it is still necessary to reinforce the three approaches for the prevention and treatment of anaemia: food diversification, drug supplementation, and food fortification, given the current prevalence of anaemia and insufficient consumption of dietary iron sources.

The main strengths of this study include an extensive data search in six databases and gray literature and a robust methodology to estimate subgroups’ prevalence by region, age, and before and after the period of implementation of public policies in Brazil. These efforts provide the actual and unpublished Brazilian anaemia prevalence and assists to support the development of new dietary strategies associated with nutritional supplementary programs to combat and prevent pregnancy anaemia. 

Among the possible limitations of this study, firstly some studies did not present all the data necessary for the analyses, and the authors did not respond to contact attempts. Second, the large heterogeneity between studies may make the analysis of the current anaemia prevalence difficult, and even the subgroups analysis was still heterogeneous and meta-regression was unable to identify the sources of this heterogeneity. Third, the number of studies published in the North, South, and Midwest regions was to the detriment of the Northeast and Southeast regions, which may have biased the subgroup’s results. To better prevent high bias of future studies, we suggested to carry out more high-quality original studies in the mentioned regions, to use national electronic patient databases and to conduct national multicentric studies in order to better describe Brazilian pregnant women’s micronutrient deficiency. Further, it is necessary to investigate several nutritional causes of anaemia beyond iron deficiency to propose more assertive health public policies. 

## 5. Conclusions

The overall prevalence of anaemia in Brazilian pregnant women was estimated to be 23%, similar to the latest data published by the WHO. Anaemia remains a moderate national public health problem and considering the side effects of anaemia in the pregnant woman and the child groups, further high-quality studies are needed to investigate the causes of anaemia, whether due to iron deficiency, vitamin B12, folic acid, chronic inflammation or other single or combined factors to ensure adequate anaemia prevention and management. Furthermore, a constant evaluation of public health actions is necessary to address the causes of maternal anaemia in Brazil. These results can be combined to promote more effective public policies mitigating health damage and combating anaemia in the maternal group.

## Figures and Tables

**Table 1:** Summary of included studies’ characteristics.

Author	Study Period	Geographic Region of Brazil	Study Design	Location of Data Collection	Age (Range or Mean)	Gestational Age (Range or Mean)	Sample Size n	Capillary or Venous	Criteria for Anemia Status	Prevalence of Anaemia (%)	95% CI	Quality Grade (Risk)
Soares et al. (2021) [56]	2020	Northeast	Cross-sectional	Primary health unit	12–45	NR	278	Venous	WHO (Hb < 11 g/dL)	26.98	22.01; 32.43	High
Figueiredo et al. (2019) [63]	2013–2017	Northeast	Cohort	Primary health unit	13–46	NR	622	Venous	WHO (Hb < 11 g/dL)	24.9	21.64; 28.43	High
Santana et al. (2019) [46]	2012	Northeast	Cross-sectional	Primary health unit	NR	NR	208	Venous	WHO (Hb < 11 g/dL)	16.8	12.2; 22.37	High
Bezerra et al. (2018) [50]	2014	Northeast	Cross-sectional	Community	14–37	1st, 2nd and 3rd Trimester	45	Venous	WHO (Hb < 11 g/dL)	7	1.72; 17.08	High
Frick et al. (2018) [29]	2009–2011	South	Cross-sectional	Primary health unit	10–49	1st Trimester	107	Venous	WHO (Hb < 11 g/dL)	14.02	8.37; 21.59	High
Magalhães et al. (2018) [34]	2010–2011	Northeast	Cross-sectional	Primary health unit	20–35	1st, 2nd and 3rd Trimester	328	Capillary	WHO (Hb < 11 g/dL)	18.9	14.94; 23.41	High
Miranda et al. (2018) [36]	2015	South	Cohort	Community	13–46	NR	3419	Venous	WHO (Hb < 11 g/dL)	35.9	34.29; 37.51	High
Neme et al. (2017) [37]	2008	South	Cross-sectional	Primary health unit	NR	NR	91	Venous	WHO (Hb < 11 g/dL)	35.16	25.88; 45.38	High
Santos (2016) [48]	2015–2016	North	Cross-sectional	Primary health unit	13–40	2nd Trimester	506	Venous	WHO (Hb < 11 g/dL)	15.4	12.3; 18.9	High
Silva (2015) [54]	2013	Northeast	Cross-sectional	Primary health unit	13–41	1st, 2nd and 3rd Trimester	349	Venous	WHO (Hb < 11 g/dL)	22.64	18.47; 27.25	High
Oliveira et al. (2015) [38]	2014	Northeast	Cross-sectional	Primary health unit	14–44	NR	428	Capillary	WHO (Hb < 11 g/dL)	28.30	24.16; 32.68	Low
Silva (2014) [53]	2013	Northeast	Cross-sectional+E11	Primary health unit	14–40	1st, 2nd and 3rd Trimester	201	Venous	WHO (Hb < 11 g/dL)	27.86	21.99; 34.36	High
Aragão et al. (2013) [27]	2011	Northeast	Cross-sectional	Hospital	15–40	1st, 2nd and 3rd Trimester	59	Venous	WHO (Hb < 11 g/dL)	20.3	11.51; 32.02	High
Camargo et al. (2013) [58]	2008–2009	Midwest	Cross-sectional	University Hospital	19–49	2nd Trimester	146	Venous	WHO (Hb < 11 g/dL)	5	2.121; 9.25	High
Marion (2013) [35]	2011	South	Cross-sectional	University Hospital	16–44	1st, 2nd and 3rd Trimester	124	Venous	WHO (Hb < 11 g/dL)	16.2	10.43; 23.4	High
Santos et al. (2012) [51]	2008	Northeast	Cross-sectional	Maternity	16–40	1st, 2nd and 3rd Trimester	118	Venous	WHO (Hb < 11 g/dL)	32.2	24.32; 41.03	High
Silva (2012) [52]	2011–2012	Northeast	Cross-sectional	Hospital	18–35	NR	611	Venous	WHO (Hb < 11 g/dL)	29.9	26.42; 33.67	High
Quintans (2011) [42]	2009	Northeast	Cross-sectional	Primary health unit	18–42	1st, 2nd and 3rd Trimester	130	Venous	WHO (Hb < 11 g/dL)	17.7	11.84; 24.97	High
Soares et al. (2010) [55]	2004–2008	Southeast	Cohort	Prenatal clinic	15–37	NR	183	Venous	WHO (Hb < 11 g/dL)	10.4	6.55; 15.45	High
Costa et al. (2009) [60]	2006	North	Cross-sectional	Primary health unit	NR	1st, 2nd and 3rd Trimester	92	Venous	WHO (Hb < 11 g/dL)	26.1	17.89; 35.77	High
Santos et al. (2009) [49]	NR	Southeast	Cross-sectional	Prenatal clinic	13–17	3rd Trimester	24	Venous	WHO (Hb < 11 g/dL)	41.6	23.45; 61.79	High
Ferreira et al. (2008) [62]	2007	Northeast	Cross-sectional	Community	16–43	1st, 2nd and 3rd Trimester	150	Capillary	WHO (Hb < 11 g/dL)	50	42.04; 57.96	High
Sakamoto (2008) [45]	2007	Midwest	Cross-sectional	University Hospital	14–42	1st, 2nd and 3rd Trimester	233	Venous	WHO (Hb < 11 g/dL)	18	13.48; 23.36	High
Paiva et al. (2007) [40]	2000	Southeast	Cross-sectional	Hospital	NR	NR	95	Venous	WHO (Hb < 11 g/dL)	19	12; 27.76	High
Côrtes (2006) [59]	2004	Midwest	Cross-sectional	University Hospital	<20, >35	1st, 2nd and 3rd Trimester	228	Capillary	WHO (Hb < 11 g/dL)	28.94	23.34; 35.09	Low
Lucyk (2006) [33]	2005	Midwest	Cross-sectional	University Hospital	NR	1st, 2nd and 3rd Trimester	170	Capillary	WHO (Hb < 11 g/dL)	19.40	13.98; 25.87	High
Santos (2006) [47]	2005–2006	Northeast	Cross-sectional	Primary health unit	12–44	1st, 2nd and 3rd Trimester	326	Venous	WHO (Hb < 11 g/dL)	31.9	27.01; 37.11	High
Rocha et al. (2005) [43]	2002–2003	Southeast	Cross-sectional	Primary health unit	14–38	1st, 2nd and 3rd Trimester	168	Capillary	WHO (Hb < 11 g/dL)	21.4	15.72; 28.12	High
Papa et al. (2003) [41]	2001–2002	Southeast	Cross-sectional	University	>16	1st and 2nd Trimester	56	Venous	WHO (Hb < 11 g/dL)	21.4	12.16; 33.59	High
Dias (2000) [61]	NR	Southeast	Cross-sectional	Primary health unit	NR	NR	36	Venous	WHO (Hb < 11 g/dL)	10	3.63; 24.66	High
Fujimori et al. (2000) [31]	2000	Southeast	Cross-sectional	Primary health unit	NR	1st, 2nd and 3rd Trimester	72	Venous	WHO (Hb < 11 g/dL)	13.9	7.27; 23.36	High
Arruda (1997) [39]	1992	Northeast	Cross-sectional	Maternity	NR	NR	1007	Venous	WHO (Hb < 11 g/dL)	30.88	28.09; 33.79	High
Fujimori (1994) [30]	1993	Southeast	Cross-sectional	Maternity	13–19	1st, 2nd and 3rd Trimester	155	Venous	WHO (Hb < 11 g/dL)	14.2	9.34; 20.37	High
Rodriguez et al. (1991) [44]	NR	Southeast	Cross-sectional	Hospital	NR	NR	691	Venous	WHO (Hb < 11 g/dL)	29.2	25.93; 32.71	High
Arruda (1990) [28]	1989	Northeast	Cross-sectional	Maternity	13–44	1st, 2nd and 3rd Trimester	710	Venous	WHO (Hb < 11 g/dL)	30.3	26.98; 33.74	High
Guerra et al. (1990) [32]	1988	Southeast	Cross-sectional	Primary health unit	14–46	1st, 2nd and 3rd Trimester	363	Venous	HURTADO et al., 1945, FINCH, 1969 (<11.6 g/dL)	12.4	9.29; 16.09	High
Szarfarc (1974) [57]	NR	Southeast	Cross-sectional	Philanthropic Unit	NR	3rd Trimester	263	Venous	Benjamin, Rauramo et al. e de Sanchez-Medal (<12 g/dL)	52.3	46.43; 58.46	High

**Table 2:** Prevalence of anaemia in Brazilian pregnant women and heterogeneity in subgroup analysis and between groups.

Variables	Number of Studies	Number of Participants	Prevalence (%)	95% CI	I^2^ (%)	*p* (Chi-Squared)	I^2^ between Groups (%)	*p* (Chi-Squared) between Groups
Sample size							95.02	0.00
≤500	30.00	5226.00	22.00	18–26	92.50	0.00		
>500	7.00	7566.00	28.00	23–33	95.80	0.00		
Age							95.02	0.00
Adolescents	4.00	271.00	19.00	10–28	65.62	0.03		
Adults and Adolescents	26.00	11,009.00	23.00	19–27	95.76	0.00		
Not Reported	7.00	1512.00	28.00	18–37	94.04	0.00		
Gestational Trimester							95.02	0.00
All	21.00	5460.00	24.00	20–27	89.07	0.00		
Only one or two	8.00	1269.00	22.00	11–33	96.27	0.00		
Not Reported	8.00	6063.00	25.00	18–31	95.80	0.00		
Region							95.02	0.00
North	2.00	598.00	17.00	14–20				
Northeast	16.00	5570.00	26.00	23–29	87.70	0.00		
Midwest	4.00	777.00	18.00	07–28	94.79	0.00		
Southeast	11.00	2106.00	22.00	14–30	94.72	0.00		
South	4.00	3741.00	25.00	12–38	95.70	0.00		
Data collection period							95.02	0.00
Before 2004	11.00	3616.00	24.00	17–30	94.89	0.00		
After 2004	26.00	9176.00	23.00	19–28	95.26	0.00		
Data collection period							95.02	0.00
Before 2011	25.00	5942.00	24.00	19–28	94.53	0.00		
After 2011	12.00	6850.00	23.00	18–28	95.18	0.00		
Hb determination method							95.02	0.00
Capillary	6.00	1472.00	27.00	20–35	90.74	0.00		
Venous	31.00	11,320.00	23.00	19–27	95.52	0.00		
Methodological quality					95.28	0.00	95.02	0.00
Low	2.00	656.00	29.00	25–32				
High	35.00	12,136.00	23.00	20–27				

**Table 3:** GRADE evidence profile for prevalence of anaemia in Brazilian pregnant women.

Outcomes	Risk of Bias ^a^	Inconsistency ^b^	Indirectness ^c^	Imprecision ^d^	Publication Bias ^e^	Certainty
Prevalence of anaemia in Brazilian pregnant women	Very serious	Very Serious	Not serious	Serious	Not serious	⊕◯◯◯ Very low

^a^. Risk of bias assessed using JBI. Downgrade 2 levels, considering inappropriate sampling method or statistical analyses in more than 75% of studies. ^b^. Heterogeneity was considered important when I^2^ presents values more than 40%. Downgrade 2 levels for inconsistency because I^2^ = 95.02. ^c^. No downgrade for indirectness because less than 25% of studies did not use valid and reliable methods for data collection. ^d^. Downgrade 1 level for imprecision because more than 75% of studies with small sample size (≤500). ^e^. Publication bias was considered when significance of *p* < 0.05. No downgrade because *p* = 0.090.

## Data Availability

Not applicable.

## Supplementary Materials

The following supporting information can be downloaded at: https://www.mdpi.com/article/10.3390/ijerph20021519/s1, Figure S1a–f: Prevalence of anaemia in Brazilians pregnant women; Figure S2: Funnel graph on the publication bias; Table S1: Search Strategy; Table S2: Risk of bias for each individual study JBI critical appraisal checklist for prevalence studies. Table S3: Prisma Checklist. Table S4: Excluded Articles and Reasons for Exclusion.
