# Supplementary material for: The Prevalence of Nutritional Anaemia in Brazilian Pregnant Women: A Systematic Review and Meta-Analysis
Source: Int J Environ Res Public Health. 2023 Jan 13;20(2):1519. doi: 10.3390/ijerph20021519 (PMC9859559; doi:10.3390/ijerph20021519)
Supplement: Supplementary file 1 [file ijerph-20-01519-s001.zip › ijerph-2091012-supplementary.docx]

Table S1. Database search strategy.

| **DATABASE** | **SEARCH** (June, 29, 2021; January, 03, 2022, May, 14, 2022 and November, 10, 2022) |
| --- | --- |
| **MEDLINE** | (((Pregnancy OR "Pregnant women" OR Gravidity OR Pregnant OR Gravid OR Antenatal OR Antepartum OR Gestation) AND (Brazil OR Brazilian)) AND (anemia OR anaemia OR haemoglobin OR hemoglobin OR haematocrit OR hematocrit OR "iron deficiency anemia" OR "iron deficiency anaemia" OR "human hemoglobin" OR "human haemoglobin" OR "hemoglobin levels" OR "haemoglobin levels" OR "Iron deficiency" OR "Iron-Deficiency Anemia" OR "Maternal anemia" OR ferritin)) AND ("Observational Study" OR "Cohort study" OR "Longitudinal study" OR "Follow-up study" OR Cohort OR Longitudinal OR Prospective OR Retrospective OR "Incidence study" OR Follow-up OR "Prevalence study" OR Prevalence OR "Cross-Sectional Study" OR Cross-Sectional OR frequency) |
| **EMBASE** | ('pregnancy'/exp OR pregnancy OR 'pregnant women'/exp OR 'pregnant women' OR 'gravidity'/exp OR gravidity OR pregnant OR gravid OR antenatal OR antepartum OR 'gestation'/exp OR gestation) AND ('brazil'/exp OR brazil OR 'brazilian'/exp OR brazilian) AND ('anemia'/exp OR anemia OR 'anaemia'/exp OR anaemia OR 'haemoglobin'/exp OR haemoglobin OR 'hemoglobin'/exp OR hemoglobin OR 'haematocrit'/exp OR haematocrit OR 'hematocrit'/exp OR hematocrit OR 'iron deficiency anemia'/exp OR 'iron deficiency anemia' OR 'iron deficiency anaemia'/exp OR 'iron deficiency anaemia' OR 'human hemoglobin' OR 'human haemoglobin' OR 'hemoglobin levels' OR 'haemoglobin levels' OR 'iron deficiency'/exp OR 'iron deficiency' OR 'iron-deficiency anemia'/exp OR 'iron-deficiency anemia' OR 'maternal anemia'/exp OR 'maternal anemia' OR 'ferritin'/exp OR ferritin) AND ('observational study'/exp OR 'observational study' OR 'cohort study'/exp OR 'cohort study' OR 'longitudinal study'/exp OR 'longitudinal study' OR 'follow-up study'/exp OR 'follow-up study' OR cohort OR 'longitudinal'/exp OR longitudinal OR prospective OR retrospective OR 'incidence study' OR 'follow up'/exp OR 'follow up' OR 'prevalence study'/exp OR 'prevalence study' OR 'prevalence'/exp OR prevalence OR 'cross-sectional study'/exp OR 'cross-sectional study' OR 'cross sectional' OR 'frequency'/exp OR frequency) |
| **LILACS** | Anemia AND pregnancy AND Brazil |
| **SCOPUS** | TITLE-ABS-KEY ( ( ( ( pregnancy OR "Pregnant women" OR gravidity OR pregnant OR gravid OR antenatal OR antepartum OR gestation ) AND ( brazil OR brazilian ) ) AND ( anemia OR anaemia OR haemoglobin OR hemoglobin OR haematocrit OR hematocrit OR "iron deficiency anemia" OR "iron deficiency anaemia" OR "human hemoglobin" OR "human haemoglobin" OR "hemoglobin levels" OR "haemoglobin levels" OR "Iron deficiency" OR "Iron-Deficiency Anemia" OR "Maternal anemia" OR ferritin ) ) AND ( "Observational Study" OR "Cohort study" OR "Longitudinal study" OR "Follow-up study" OR cohort OR longitudinal OR prospective OR retrospective OR "Incidence study" OR follow-up OR "Prevalence study" OR prevalence OR "Cross-Sectional Study" OR cross-sectional OR frequency ) ) |
| **WEB OF SCIENCE** | (((Pregnancy OR "Pregnant women" OR Gravidity OR Pregnant OR Gravid OR Antenatal OR Antepartum OR Gestation) AND (Brazil OR Brazilian)) AND (anemia OR anaemia OR haemoglobin OR hemoglobin OR haematocrit OR hematocrit OR "iron deficiency anemia" OR "iron deficiency anaemia" OR "human hemoglobin" OR "human haemoglobin" OR "hemoglobin levels" OR "haemoglobin levels" OR "Iron deficiency" OR "Iron-Deficiency Anemia" OR "Maternal anemia" OR ferritin)) AND ("Observational Study" OR "Cohort study" OR "Longitudinal study" OR "Follow-up study" OR Cohort OR Longitudinal OR Prospective OR Retrospective OR "Incidence study" OR Follow-up OR "Prevalence study" OR Prevalence OR "Cross-Sectional Study" OR Cross-Sectional OR frequency) |
| **GOOGLE SCHOLAR** | Pregnancy OR Pregnancies OR Gestation AND Brazil OR Brazilian AND Anemia OR Anaemia OR hematocrit OR haematocrit OR hemoglobin OR haemoglobin AND Prevalence OR "Observational Study" OR "Longitudinal study" OR "Incidence study" OR "Prevalence study" |
| **GOOGLE ACADÊMICO** | noft(Pregnancy OR Pregnancies OR Gestation OR Pregnant OR "Pregnant Women" OR "pregnant woman" OR "Prenatal Care" OR "Pregnancy in Adolescence" OR "Teen Pregnancy" OR "Teen Pregnancies" OR "Adolescent Pregnancy" OR "Adolescent Pregnancies") AND noft("Iodine deficiency" OR "Iodine insufficiency" OR "Iodine status" OR "Urinary Iodine Concentration" OR "serum iodine" OR iodine OR "iodine intake") AND noft(Prevalence OR Frequency OR Percentage OR Percent OR Proportion OR Ratio OR Rate) AND noft(Survey OR "Cross-sectional studies" OR "Cross-sectional" OR Observational OR Cohort) |
| **SCIELO** | gestação and anemia |
| **CATÁLOGO DE TESES E DISSERTAÇÕES DA CAPES** | gestantes AND anemia |

**Table S2.** Risk of bias for each individual study assessed by Joanna Briggs Institute critical appraisal checklist for prevalence studies.

| **Studies** | **Criteria** | | | | | | | | | |
| --- | --- | --- | --- | --- | --- | --- | --- | --- | --- | --- |
|  | **1*** | **2*** | **3*** | **4*** | **5*** | **6*** | **7*** | **8*** | **9*** |  |
| **Aragão et al, 2013 [74]** | Y | N | N | Y | Y | U | U | N | Y |  |
| **Arruda, 1990 [75]** | Y | N | Y | Y | Y | Y | Y | N | Y |  |
| **Arruda, 1997 [86]** | Y | N | Y | Y | Y | Y | Y | N | Y |  |
| **Bezerra et al, 2018 [97]** | Y | N | N | Y | Y | Y | Y | N | Y |  |
| **Camargo et al, 2013 [105]** | Y | N | N | Y | Y | Y | Y | Y | Y |  |
| **Côrtes, 2006 [106]** | Y | Y | Y | Y | Y | Y | Y | Y | Y |  |
| **Costa et al, 2009 [107]** | N | N | N | N | Y | Y | Y | N | Y |  |
| **Dias, 2000 [108]** | Y | N | N | Y | Y | Y | Y | N | Y |  |
| **Ferreira et al, 2008 [109]** | Y | Y | Y | Y | Y | Y | Y | N | Y |  |
| **Figueiredo et al, 2019 [110]** | Y | Y | Y | Y | Y | Y | Y | N | Y |  |
| **Frick et al, 2018 [76]** | Y | N | N | Y | Y | Y | Y | N | Y |  |
| **Fujimori et al, 2000 [78]** | Y | N | N | Y | Y | Y | Y | N | Y |  |
| **Fujimori, 1994 [77]** | Y | N | N | Y | Y | Y | Y | N | Y |  |
| **Guerra et al, 1990 [79]** | Y | N | Y | Y | Y | Y | Y | N | Y |  |
| **Lucyk, 2006 [80]** | Y | N | N | Y | Y | Y | Y | N | Y |  |
| **Magalhães et al, 2018 [81]** | Y | Y | Y | Y | Y | Y | Y | N | Y |  |
| **Marion, 2013 [82]** | Y | N | N | Y | Y | Y | Y | N | Y |  |
| **Miranda et al, 2018 [83]** | Y | U | Y | Y | Y | Y | Y | Y | Y |  |
| **Neme et al, 2017 [84]** | Y | N | N | N | Y | Y | N | N | Y |  |
| **Oliveira et al, 2015 [85]** | Y | Y | Y | Y | Y | Y | Y | Y | Y |  |
| **Paiva et al, 2007 [87]** | Y | N | Y | N | Y | Y | Y | N | Y |  |
| **Papa et al, 2003 [88]** | Y | N | N | Y | Y | Y | Y | N | Y |  |
| **Quintans, 2011 [89]** | Y | N | N | N | Y | Y | Y | N | Y |  |
| **Rocha et al, 2005 [90]** | Y | N | Y | Y | Y | Y | Y | N | Y |  |
| **Rodriguez et al, 1991 [91]** | Y | N | N | N | Y | Y | Y | N | Y |  |
| **Sakamoto, 2008 [92]** | Y | N | N | Y | Y | Y | Y | N | Y |  |
| **Santana et al, 2019 [93]** | Y | N | N | Y | Y | Y | Y | Y | Y |  |
| **Santos et al, 2009 [96]** | Y | N | N | N | Y | Y | Y | N | Y |  |
| **Santos et al, 2012 [66]** | Y | Y | Y | Y | Y | Y | Y | N | Y |  |
| **Santos, 2006 [94]** | Y | N | Y | Y | Y | Y | Y | N | Y |  |
| **Santos, 2016 [95]** | Y | N | Y | Y | Y | Y | Y | N | Y |  |
| **Silva, 2012 [99]** | Y | N | Y | Y | Y | Y | Y | N | Y |  |
| **Silva, 2014 [100]** | Y | N | Y | Y | Y | Y | Y | Y | Y |  |
| **Silva, 2015 [101]** | Y | N | Y | Y | Y | Y | Y | N | Y |  |
| **Soares et al, 2010 [102]** | Y | Y | Y | Y | Y | Y | Y | N | Y |  |
| **Soares et al, 2021 [103]** | Y | N | Y | N | Y | Y | Y | N | Y |  |
| **Szarfarc, 1974 [104]** | Y | N | N | N | Y | Y | Y | N | Y |  |

Y = Yes, N = No, U = Unclear, NA = Not applicable

1* The sample frame appropriate to address the target population

2* Criteria for sampled in an appropriate way

3* Adequate sample size

4* Study subjects and the setting described in detail

5* Analysis conducted with sufficient coverage of the identified sample

6* Outcomes measured in a valid way

7* Objective and standard criteria for measurement

8* Appropriate statistical analysis

9* Strategies for dealing with the response rate properly

Table S3 – **PRISMA CHECKLIST**

| **Section and Topic** | **Item #** | **Checklist item** | **Location**  **where item**  **is reported** |
| --- | --- | --- | --- |
| **TITLE** | | |  |
| Title | 1 | Identify the report as a systematic review. | 1 |
| **ABSTRACT** | | |  |
| Abstract | 2 | See the PRISMA 2020 for Abstracts checklist. | 1 |
| **INTRODUCTION** | | |  |
| Rationale | 3 | Describe the rationale for the review in the context of existing knowledge | 1-2 |
| Objectives | 4 | Provide an explicit statement of the objective(s) or question(s) the review addresses. | 2 |
| **METHODS** | | |  |
| Eligibility criteria | 5 | Specify the inclusion and exclusion criteria for the review and how studies were grouped for the syntheses | 2 |
| Information sources | 6 | Specify all databases, registers, websites, organisations, reference lists and other sources searched or consulted to identify studies. Specify the date when each source was last searched or consulted. | 3 |
| Search strategy | 7 | Present the full search strategies for all databases, registers and websites, including any filters and limits used. | 3, Supplementary Table S1 |
| Selection process | 8 | Specify the methods used to decide whether a study met the inclusion criteria of the review, including how many reviewers screened each record and each report retrieved, whether they worked independently, and if applicable, details of automation tools used in the process. | 3-4, figure 1, and Supplementary Appendix B |
| Data collection process | 9 | Specify the methods used to collect data from reports, including how many reviewers collected data from each report, whether they worked independently, any processes for obtaining or confirming data from study investigators, and if applicable, details of automation tools used in the process. | 3-4 |
| Data items | 10a | List and define all outcomes for which data were sought. Specify whether all results that were compatible with each outcome domain in each study were sought (e.g. for all measures, time points, analyses), and if not, the methods used to decide which results to collect. | 3-4 |
|  | 10b | List and define all other variables for which data were sought (e.g. participant and intervention characteristics, funding sources). Describe any assumptions made about any missing or unclear information. | 3-4 |
| Study risk of bias Assessment | 11 | Specify the methods used to assess risk of bias in the included studies, including details of the tool(s) used, how many reviewers assessed each study and whether they worked independently, and if applicable, details of automation tools used in the process. | 3-4 |

| **Section and Topic** | **Item #** | **Checklist item** | **Location**  **where item**  **is reported** |
| --- | --- | --- | --- |
| Effect measures | 12 | Specify for each outcome the effect measure(s) (e.g. risk ratio, mean difference) used in the synthesis or presentation of results. | 3-4 |
| Synthesis methods | 13a | Describe the processes used to decide which studies were eligible for each synthesis (e.g. tabulating the study intervention characteristics and comparing against the planned groups for each synthesis (item #5)). | 3-4 |
|  | 13b | Describe any methods required to prepare the data for presentation or synthesis, such as handling of missing summary statistics, or data conversions | 4 |
|  | 13c | Describe any methods used to tabulate or visually display results of individual studies and syntheses. | 4 |
|  | 13d | Describe any methods used to synthesize results and provide a rationale for the choice(s). If meta-analysis was performed, describe the model(s), method(s) to identify the presence and extent of statistical heterogeneity, and software package(s) used. | 4 |
|  | 13e | Describe any methods used to explore possible causes of heterogeneity among study results (e.g. subgroup analysis, meta-regression). | 4 |
|  | 13f | Describe any sensitivity analyses conducted to assess robustness of the synthesized results. | 4 |
| Reporting bias assessment | 14 | Describe any methods used to assess risk of bias due to missing results in a synthesis (arising from reporting biases). | 4 |
| Certainty Assessment | 15 | Describe any methods used to assess certainty (or confidence) in the body of evidence for an outcome | 4 |
| **RESULTS** | | |  |
| Study selection | 16a | Describe the results of the search and selection process, from the number of records identified in the search to the number of studies included in the review, ideally using a flow diagram. | 4-5 |
|  | 16b | Cite studies that might appear to meet the inclusion criteria, but which were excluded, and explain why they were excluded. | 5, Appendix B |
| Study characteristics | 17 | Cite each included study and present its characteristics. | 5-10, Table 1 |
| Risk of bias in studies | 18 | Present assessments of risk of bias for each included study. | 10, Figure 2, Table S1 |
| Results of individual studies | 19 | For all outcomes, present, for each study: (a) summary statistics for each group (where appropriate) and (b) an effect estimate and its precision (e.g. confidence/credible interval), ideally using structured tables or plots. | 11-12 |
| Results of syntheses | 20a | For each synthesis, briefly summarise the characteristics and risk of bias among contributing studies. | 11-12, Table 1 |
|  | 20b | Present results of all statistical syntheses conducted. If meta-analysis was done, present for each the summary estimate and its precision (e.g. confidence/credible interval) and measures of statistical heterogeneity. If comparing groups, describe the direction of the effect. | 12-13, and Figure 2 |

| **Section and Topic** | | **Item #** | **Checklist item** | **Location**  **where item**  **is reported** |
| --- | --- | --- | --- | --- |
|  | | 20c | Present results of all investigations of possible causes of heterogeneity among study results. | 11 |
|  | | 20d | Present results of all sensitivity analyses conducted to assess the robustness of the synthesized results. | 11-12, and Table 1 |
| Reporting biases | | 21 | Present assessments of risk of bias due to missing results (arising from reporting biases) for each synthesis assessed. | Table S2 |
| Certainty of evidence | | 22 | Present assessments of certainty (or confidence) in the body of evidence for each outcome assessed. | Table 1 |
| **DISCUSSION** | | | |  |
| Discussion |  | 23a | Provide a general interpretation of the results in the context of other evidence. | 13-14 |
|  | | 23b | Discuss any limitations of the evidence included in the review. | 16, Table 3 |
|  | | 23c | Discuss any limitations of the review processes used. | 16 |
|  | | 23d | Discuss implications of the results for practice, policy, and future research. | 16 |
| **OTHER INFORMATION** | | | | |
| Registration and protocol | | 24a | Provide registration information for the review, including register name and registration number, or state that the review was not registered. | 2 |
|  | | 24b | Indicate where the review protocol can be accessed, or state that a protocol was not prepared. | 2 |
|  | | 24c | Describe and explain any amendments to information provided at registration or in the protocol. | 2 |
| Support | | 25 | Describe sources of financial or non-financial support for the review, and the role of the funders or sponsors in the review. | 17 |
| Competing interests | | 26 | Declare any competing interests of review authors. | 17 |
| Availability of data, code and  other materials | | 27 | Report which of the following are publicly available and where they can be found: template data collection forms; data extracted from included studies; data used for all analyses; analytic code; any other materials used in the review. | NA |

*From: Page MJ, McKenzie JE, Bossuyt PM, Boutron I, Hoffmann TC, Mulrow CD, et al. The PRISMA 2020 statement: an updated guideline for reporting systematic reviews. BMJ 2021;372:n71. doi: 10.1136/bmj.n71*

Table S4 - **Excluded articles and reasons for exclusion**

| **Author, Year, Reference** | **Reason for exclusion** |
| --- | --- |
| Abreu, 2009[1]; Araújo, 2007[2]; Araújo, 2013[3]; Betiol, 2005[4]; Bresani, 2007[5]; Bresani, 2009[6]; Filho, 2011[7]; Fujimori, 2011[8]; Fujimori, 2009[9]; Guia, 2021[10]; Land, 2018[11]; Machado, 2011[12]; Machado, 2016[13]; Massucheti, 2007[14]; Mattos, 2007[15]; Paula, 2016[16]; Paz, 2019[17]; Pessoa, 2015[18]; Pizzol, 2009[19]; Pompeu, 2016[20]; Pompeu, 2016[21]; Porto, 2008[22]; Santana, 2021[23]; Santos, 2019[24]; Santos, 2009[25]; Santos, 2009[26]; Sato, 2013[27]; Sato, 2010[28]; Sato, 2014[29]; Sato, 2008[30]; Sato, 2015[31]; Silva, 2010[32]; Silva, 2012[33]; Sousa, 2020[34]; Souza, 2016[35]; Surita, 2011[36]; Szarfarc, 1985[37]; Totti, 2009[38]; Vieira, 2008[39]. | 1 |
| Batista, 1988[40]; Bressane, 2004[41]; Cavalcante, 2009[42]; Lins, 2001[43]; Papa, 2002[44]; Pinto, 1975[45]; Romani, 1984[46]; Salzano, 1980[47]; Santos, 2021[48]; Souza, 2018[49]; Szarfarc, 1997[50]. | 2 |
| Almeida, 2019[51]; Figueiredo, 2017[52]; Malta, 2016[53]; Mocelin, 2019[54]; Sato, 2011[55]; Sato, 2012[56]; Sato, 2012[57]. | 3 |
| Barbosa, 2011[58]; Borges, 2016[59]; Cavalcante, 2011[60]; Salvi, 2014[61]; Szarfarc, 1983[62]. | 4 |
| Demétrio, 2017[63]; Lima, 2021[64]; Silva, 2020[65] Santos 2012[66]. | 5 |
| Faria, 2007[67]; Maffessoni, 2021[68]. | 6 |
| Ferracioli, 2017[69]. | 7 |
| Novaes, 2015[70]. | 8 |
| Meier, 2003[71]. | 9 |
| Rodrigues,2010[72]. | 10 |
| Cruz, 2009[73]. | 11 |

Legend: (1) Use secondary data; (2) Text not available for full reading and author did not respond; (3) Abstract of Congress or Seminar; (4) No prevalence data; (5) Article uses cohort data already included in the review; (6) The anemia classification criteria is not mentioned; (7) Anemia only reported by the pregnant woman; (8) Study with puerperal women only; (9) Study carried out outside Brazil; (10) Literature Review; (11) Author did not respond with additional information.

Figures S1a to S1f - Prevalence of anaemia in Brazilians pregnant women by subgroups:


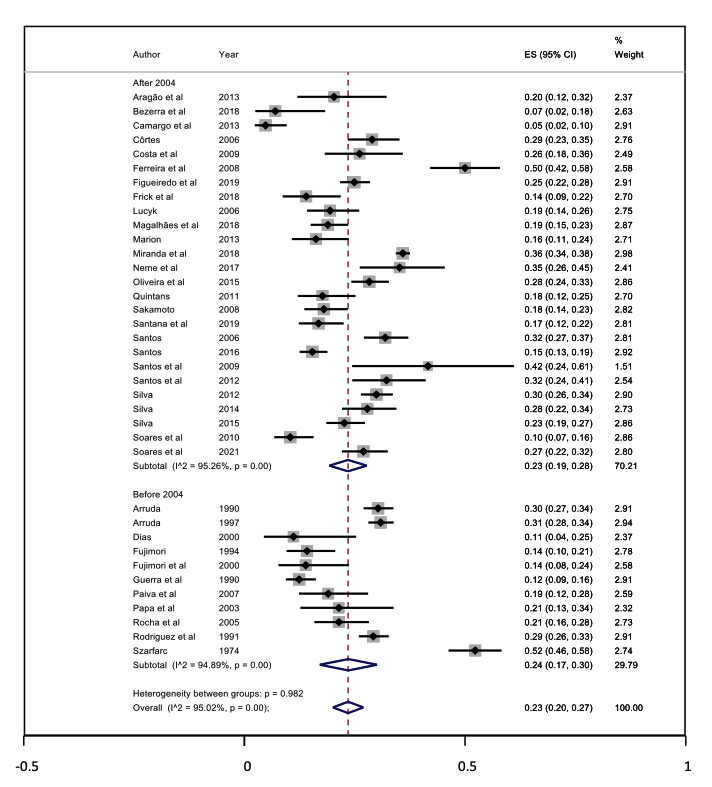


**Figure S1 a -** Prevalence of anaemia in Brazilians pregnant women – before and after 2004 [74-110]


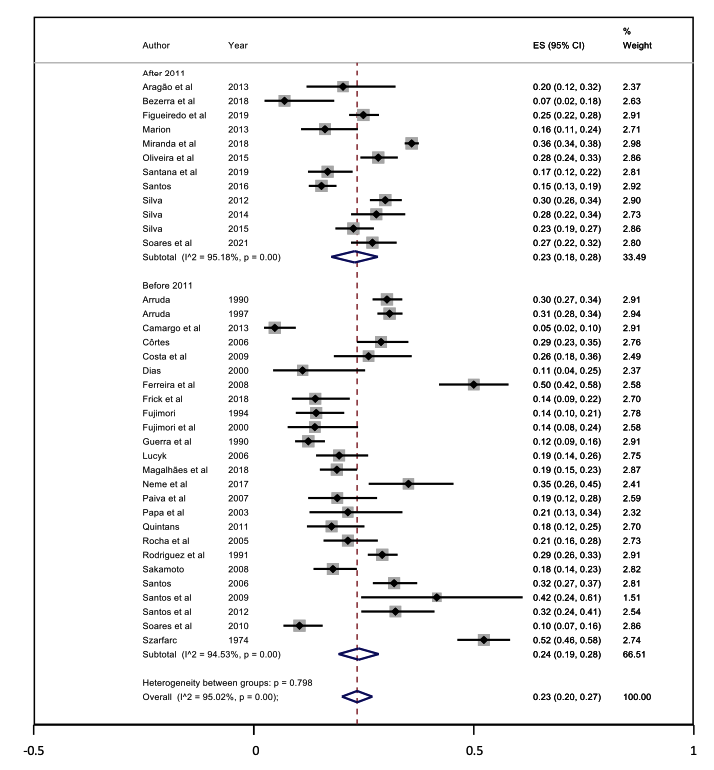


**Figure S1 b -** Prevalence of anaemia in Brazilians pregnant women – before and after 2011 [74-110]


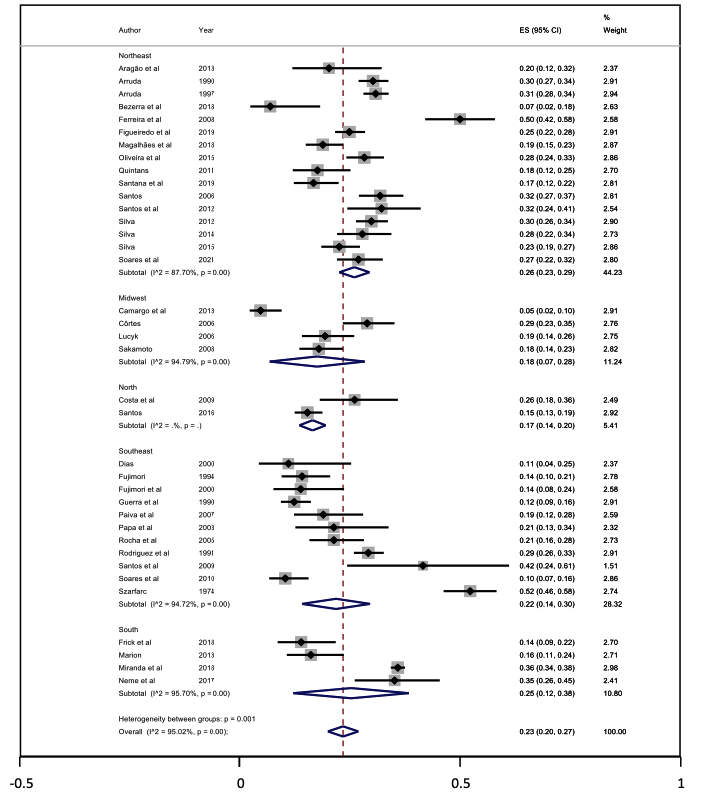


**Figure S1 c -** Prevalence of anaemia in Brazilians pregnant women – Geographic Region [74-110]


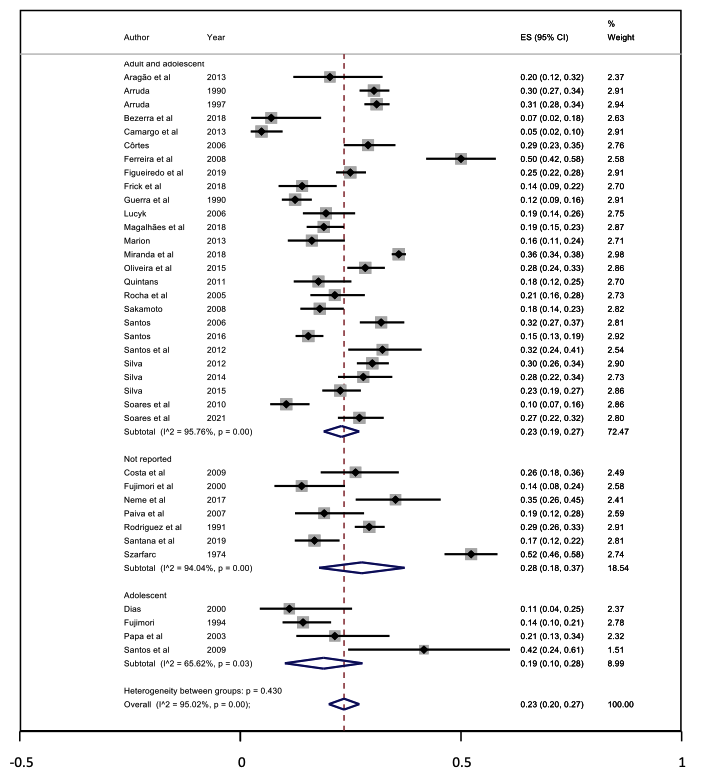


**Figure S1 d -** Prevalence of anaemia in Brazilians pregnant women – Course of Life [74-110]


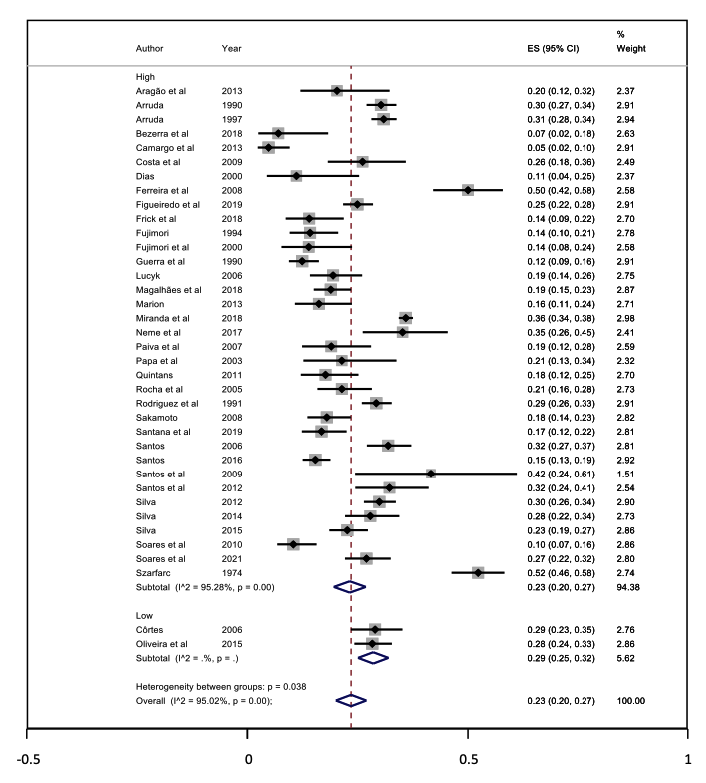


**Figure S1 e -** Prevalence of anaemia in Brazilians pregnant women – Risk of Bias [74-110]


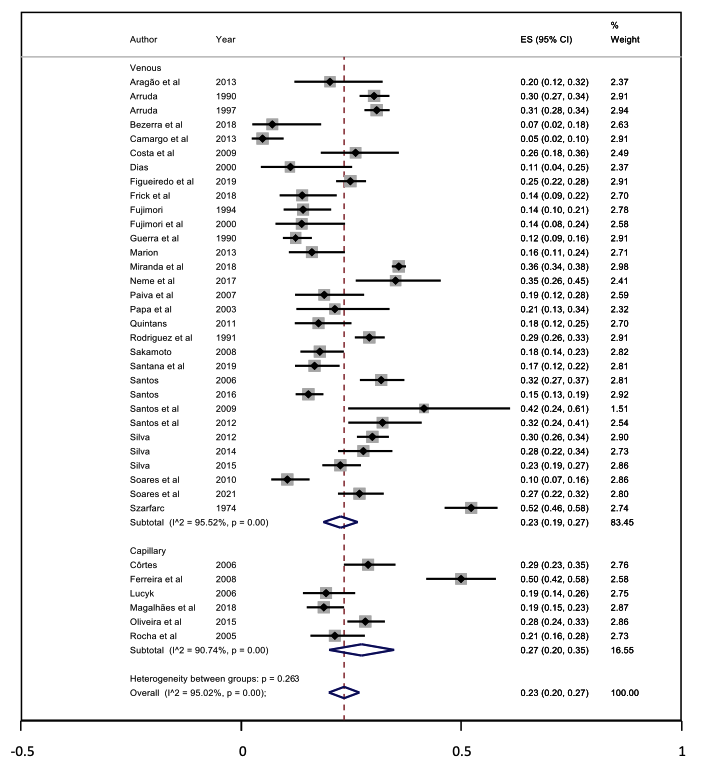


**Figure S1 f -** Prevalence of anaemia in Brazilians pregnant women – Hb determination method [74-110]

**
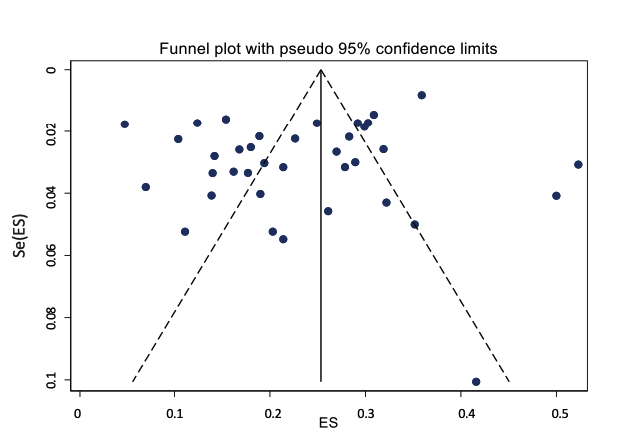
**

**Figure S2 -** Funnel graph on the publication bias.

REFERÊNCIAS

1. Abreu, L.C. Impacto Da Fortificação Das Farinhas Com Ferro, No Controle Da Anemia Em Gestantes: Estudo Em Um Serviço Público de Saúde Do Município de São Bernardo Do Campo. **2009**.

2. Araújo, C.R.M.A. Concentração de Hemoglobina em Gestantes Atendidas em Serviços de Saúde do Sul do Brasil, Antes e Após a Ingestão de Ferro em Alimentos Fortificados, Universidade Estadual de Maringá - UEM, 2007.

3. Araújo, C.R.M.A.; Uchimura, T.T.; Fujimori, E.; Nishida, F.S.; Veloso, G.B.L.; Szarfarc, S.C. Níveis de Hemoglobina e Prevalência de Anemia Em Gestantes Atendidas Em Unidades Básicas de Saúde, Antes e Após a Fortificação Das Farinhas Com Ferro. *Rev. Bras. Epidemiol.* **2013**, *16*, 535–545, doi:10.1590/S1415-790X2013000200027.

4. Betiol, M.T. Anemia Em Mulheres Grávidas Assistidas Pelo SUS No Município de Irati - PR 2004 Anemia Em Mulheres Grávidas Assistidas Pelo SUS No Município de Irati - PR 2004. **2005**.

5. Bresani, C.C.; De Souza, A.I.; Batista Filho, M.; Figueiroa, J.N. Anemia e Ferropenia Em Gestantes: Dissensos de Resultados de Um Estudo Transversal. *Rev. Bras. Saude Matern. Infant.* **2007**, *7*, 15–21, doi:10.1590/s1519-38292007000600002.

6. Bresani, C.C.; Souza, A.I.; Batista Filho, M. Erythrocyte Indices in the Second Trimester of Pregnancy: Are Reference Values Well Established? *Rev. Bras. Hematol. Hemoter.* **2009**, *31*, 37–40, doi:10.1590/s1516-84842009005000007.

7. Filho, M.D. de S.; Damasceno, C.V.X.; Szarfarc, S.C.; Fujimori, E.; Araújo, M.A. de M.; Moreira-Araújo, R.S. dos R. Fortificação Das Farinhas Com Ferro e Controle Da Anemia Em Gestantes de Teresina, Piauí, Brasil. *Rev. Nutr.* **2011**, *24*, 679–688, doi:10.1590/S1415-52732011000500002.

8. Fujimori, E.; Sato, A.P.S.; Szarfarc, S.C.; Veiga, G.V. da; Oliveira, V.A. de; Colli, C.; Moreira-Araújo, R.S. dos R. Anemia Em Gestantes Brasileiras Antes e Após a Fortifi Cação Das Farinhas Com Ferro Anemia in Brazilian Pregnant Women before and after Fl Our Fortifi Cation. *Rev. Saúde Pública 2011* **2011**, *45*, 1027–1035.

9. Fujimori, E.; Sato, A.P.S.; Araújo, C.R.M.A.; Uchimura, T.T.; Porto, E. da S.; Brunken, G.S.; Borges, A.L.V.; Szarfarc, S.C. Anemia in Pregnant Women from Two Cities in the South and Mid-West Regions of Brazil. *Rev. da Esc. Enferm.* **2009**, *43*, 1204–1209, doi:10.1590/S0080-62342009000600010.

10. Guia, L. de C.M.; Lessa, E.M.; Mesquita, T.L.; Queiroz, L.C. de; Oliveira, I.V. de; Pereira, T.M.; Lima, G.B.; Pannain, G.D.; Menon, C. de O.; Zimmermmann, J.B. Frequência de Anemia: Uma Comparação Entre Gestantes Adolescentes e Adultas. *Rev. Eletrônica Acervo Saúde* **2021**, *13*, e8417, doi:10.25248/reas.e8417.2021.

11. Land, A.; Hoffmann Palú, F. Perfil Socioeconômico E Hematológico De Gestantes Atendidas Na Unidade Básica De Saúde Do Município De Guaraciaba, Santa Catarina. *Core.Ac.Uk* **2018**, 83–90.

12. Machado, E.H. da S. Anemia Em Gestantes Atendidas Em Unidades Básicas de Saúde Da Região Administrativa Do Butantã, Município de São Paulo, Em 2006 e 2008. **2011**, 71.

13. Machado, E.H. da S.; Carli, E.; Szarfarc, S.C.; Souza, J.M.P.; Fujimori, E.; Colli, C. Anemia among Pregnant Women Attendingprimary Healthcare Units in the Municipalityof São Paulo, Brazil- Evaluations after Themandatory Fortification of Wheat and Maizeflours with Iron.Pdf 2016.

14. Massucheti, L. Prevalência de Anemia Em Gestantes Atendidas na Rede Pública de Saúde do Município de Florianópolis - SC, Universidade Federal de Santa Catarina, 2007, Vol. вы12у.

15. Mattos, K.M. Prevalência de Anemia Ferropriva em Gestantes Atendidas em Um Serviço De Atenção Primária, Universidade Luterana do Brasil, 2007.

16. Paula, W.K.A.S. de; Gomes, E.A. da S.; Silva, I.C. da Prevalência De Anemia Em Gestantes Acompanhadas Nas Unidades Básicas De Saúde Do Município De Caruaru-Pe. *DEMETRA Aliment. Nutr. Saúde* **2016**, *11*, 415–426, doi:10.12957/demetra.2016.17431.

17. Paz, V.M.M. Efetividade da Fortificação Das Farinhas De Trigo e Milho Com Ferro e Ácido Fólico no Controle de Anemia Em Gestantes em uma Maternidade de Referência De Teresina -PI, Universidade Federal do Piauí, 2019.

18. Pessoa, L. da S.; Saunders, C.; Belfort, G.P.; da Silva, L.B.G.; Veras, L.S.; dos Santos Esteves, A.P.V. Temporal Evolution of Anemia Prevalence in Pregnant Adolescents of a Public Maternity of Rio de Janeiro. *Rev. Bras. Ginecol. e Obstet.* **2015**, *37*, 208–215, doi:10.1590/SO100-720320150005321.

19. Dal Pizzol, T.D.S.; Giugliani, E.R.J.; Mengue, S.S. Association between Iron Supplementation during Pregnancy and Prematurity, Low Birth Weight, and Very Low Birth Weight. *Cad. Saude Publica* **2009**, *25*, 160–168, doi:10.1590/s0102-311x2009000100017.

20. Pompeu, M.P.; Surita, F.G.; Pastore, D.A.; Paulino, D.S.M.; Pinto e Silva, J.L. Anemia in Pregnant Adolescents: Impact of Treatment on Perinatal Outcomes. *J. Matern. Neonatal Med.* **2017**, *30*, 1158–1162, doi:10.1080/14767058.2016.1205032.

21. Pompeu, M.P. PERFIL NUTRICIONAL E PREVALÊNCIA DE ANEMIA EM GESTANTES ADOLESCENTES, Universidade Estadual de Campinas, 2016, Vol. 제13집 1호.

22. Porto, E. da S. Influência Da Fortificação Das Farinhas de Trigo e de Milho Com Ferro e Ácido Fólico Na Concentração de Hemoglobina de Gestantes, Universidade Federal de Mato Grosso, 2008.

23. Santana, R.B.B.; Barbosa, M.C.R.; Do Carmo, C.N.; Da Costa, R. de S.S.; Chagas, C.B.; Santos, M.S. dos; Da Silva, L.B.G.; Saunders, C. A Relação Entre as Concentrações de Hemoglobina Durante a Gravidez e Peso de Nascimento / The Relationship between Hemoglobin Concentrations during Pregnancy and Birth Weight. *Brazilian J. Dev.* **2021**, *7*, 81611–81629, doi:10.34117/bjdv7n8-396.

24. Santos, A. de J. Relação Entre Anemia Ferropriva e Condições Socioeconômicas de Gestantes em um Município do Recôncavo da Bahia, 2019, Vol. 3.

25. SANTOS, A.U.; Prevalência de Anemia em Gestantes Atendidas em uma Maternidade Social: Antes e Após a Fortificação das Farinhas com Ferro. **2009**, *2*, 255.

26. Santos, T.F.; Gkuidine, A.T.; Corrêa, J.O. do A.; Mesquita, H.L. Evaluation of Pregnant Women With Monitoring of Hemoglobin in a Low-Basic Health Unit of the State of São Paulo. *Revista* **2009**, 105–109.

27. Sato, A.P.S. Avaliação Dos Níveis de Hemoglobina de Gestantes Brasileiras Antes e Após a Fortificação de Farinhas com Ferro. **2013**, 1–10.

28. Sato, A.P.S. Anemia em Gestantes Atendidas em Serviços Públicos de Pré-Natal das Cinco Regiões Brasileiras Antes e Após a Política de Fortificação das Farinhas com Ferro. **2010**, 1–12.

29. Sato, A.P.S.; Fujimori, E.; Szarfarc, S.C. Curvas de Hemoglobina Ao Longo Da Gestação Antes e Após a Fortificação de Farinhas Com Ferro. *Rev. da Esc. Enferm.* **2014**, *48*, 409–414, doi:10.1590/S0080-623420140000300004.

30. Sato, A.P.S.; Fujimori, E.; Szarfarc, S.C.; Sato, J.R.; Bonadio, I.C. Prevalência de Anemia Em Gestantes e a Fortificação de Farinhas Com Ferro. *Texto Context. - Enferm.* **2008**, *17*, 474–481, doi:10.1590/S0104-07072008000300008.

31. Sato, A.P.S.; Porto, E.; Brunken, G.S.; Fujimori, E.; Leone, C.; Szarfarc, S.C. Anemia e Nível de Hemoglobina Em Gestantes de Cuiabá, Mato Grosso, Brasil, Antes e Após a Fortificação Compulsória de Farinhas Com Ferro e Ácido Fólico, 2003-2006. *Epidemiol. e Serviços Saúde* **2015**, *24*, 453–464, doi:10.5123/s1679-49742015000300011.

32. Silva, A.A. Condições Nutricionais de GEstantes de Risco Atendidas Em Um Ambulatório Escola. **2010**, 1–12.

33. Da Silva, C.L.; Saunders, C.; Szarfarc, S.C.; Fujimori, E.; Da Veiga, G.V. Anaemia in Pregnant Women before and after the Mandatory Fortification of Wheat and Corn Flours with Iron. *Public Health Nutr.* **2012**, *15*, 1802–1809, doi:10.1017/S1368980012001206.

34. Sousa, M.R.P.; Santos, M.B.L.; Júnior, R.N.C.M.; Araújo, E.T.H.; Silva, I.S. Diagnosis of Anemia among Pregnant Adolescents: A Document Analysis. *Angew. Chemie Int. Ed. 6(11), 951–952.* **2020**, 10–27.

35. Souza, T. Perfil Nutricional De Gestantes Adolescentes E Adultas Em Assistência Pré-Natal Em Uma Unidade Básica De Saúde De Cuiabá-Mt, Universidade Federal De Mato Grosso, 2016.

36. Surita, F.G.C.; Suarez, M.B.B.; Siani, D.S.; Pinto e Silva, J.L. Fatores Associados Ao Baixo Peso Ao Nascimento Entre Adolescentes No Sudeste Do Brasil. *Rev. Bras. Ginecol. e Obstet.* **2011**, *33*, 286–291.

37. Szarfarc, S.C. A Anemia Nutricional Entre Gestantes Atendidas Em Centros de Saúde Do Estado de São Paulo (Brasil). *Rev. Saude Publica* **1985**, *19*, 450–457, doi:10.1590/s0034-89101985000500009.

38. Totti, H.K. da S.B.; Zimmermmann, J.B.; Pena, D.M.F.; Pereira, M.P.; Bittencourt, C.; Coutinho, T. *Frequência de Anemia e Valores de Normalidade Para a Hemoglobina Em Gestantes*;

39. Vieira, M.A.G. Enteroparasitoses e Anemia Ferropriva em Gestantes Assistidas na Unidade Saúde da Família de Nova Viçosa e Posses, no Município de Viçosa-MG Dissertação. *Univ. Fed. Viçosa* **2009**.

40. Filho, M.B. Anemia Em Adolescentes Gestantes No Brasil. **1988**.

41. Bressane Prevalência de Anemia Ferropriva em Gestantes Adolescentes do Programa de Pré-Natal do PAM - Codajás - Manaus, AM. **2004**.

42. Cavalcante Estudo da Anemia e do Perfil do Ferro Em Gestantes Adolescentes Atendidas no Programa de Saúde da Família (PSF) em Iguatu. **2009**.

43. Lins Avaliacao Epidemiologica Da Gestacao Em Adolescentes de Joao Pessoa-Paraiba-Brasil. **2001**.

44. Papa, A.C.E. A Anemia Por Deficiência de Ferro de Sua Absorção Em Gestantes Adolescentes. **2002**.

45. Pinto Anemia in Pregnant Women of Sobradinho, a Satellite City of Brazilia, Brazil. **1975**.

46. Romani Anemia in Pregnant Women at 2 Health Centers of the City of Recife, PE. **1984**.

47. Salzano Prevalence of Anaemia among Pregnant Women in Two States of North East Brazil. **1980**.

48. Santos Prevalência de Anemia em Gestantes, Fatores Associados e Desfechos Perinatais de Acordo com Dois Critérios de Avaliação (OMS VERSUS CDC). **2021**.

49. Souza Study of the Prevalence of Gestational Anemia in a Public Maternity Hospital in Southern Brazil. **2018**.

50. Szarfarc, S.C. Prevalence and Risk Factors in Iron Deficiency and Anemia. **1997**.

51. Almeida Perfil Hematológico na Gestação. **2019**.

52. Figueiredo, A.C.M.G. Maternal Anemia, Health Status, Lifestyle Ame Sociodemographic Factors. **2017**.

53. Malta Gestational Weight Gain and Nutritional Status at Mid-Pregnancy in Brazilian Amazon. **2016**.

54. Mocelin ANEMIA NA GESTAÇÃO- PREVALÊNCIA E PERFIL HEMATOLÓGICO EM UM LABORATÓRIO ESCOLA. **2019**.

55. Sayuri Sato, A.P. Anaemia in Pregnant Women Assisted by Public Healthcare Services Of The Five Brazilian Regions Before And After The Policy Of Fortification Of Flours With Iron. **2011**.

56. Sayuri Sato, A.P. Hemoglobin Level in Pregnant Women Assisted by Public Prenatal Services in the Five Regions of Brazil. **2012**.

57. Sayuri Sato, A.P. Hemoglobin Level in Brazilian Pregnant Women According to Gestacional Age. **2012**.

58. Barbosa, L.; Ribeiro, D. de Q.; de Faria, F.C.; Nobre, L.N.; Lessa, A.D.C. Fatores Associados Ao Uso de Suplemento de Ácido Fólico Durante a Gestação. *Rev. Bras. Ginecol. e Obstet.* **2011**, *33*, 246–251.

59. Borges, M.C.; Buffarini, R.; Santos, R. V; Cardoso, A.M.; Welch, J.R.; Garnelo, L.; Coimbra, C.E.A.; Horta, B.L. Anemia among Indigenous Women in Brazil: Findings from the First National Survey of Indigenous People’s Health and Nutrition. *BMC Womens. Health* **2016**, *16*, doi:10.1186/s12905-016-0287-5.

60. Cavalcante, S. de O.; Dotto, L.M.G.; Koifman, S.; Cunha, M. de A.; Oliveira, M.F. de S.; Mamede, M.V.; Muniz, P.T. Atenção Pré-Natal No Município De Rio Branco, Acre- Inquérito De Base Populacional, 2007-2008.Pdf 2011.

61. Salvi, C.C.B.; Braga, M.C.; Batista Filho, M. Diagnostic Accuracy of Hemoglobin for Iron Deficiency in Pregnancy: Disclosing Results of a Cited Clinical Trial^ien. *Rev. panam. salud p�blica* **2014**, *36*, 110–116.

62. Szarfarc, S.C.; Siqueira, A.A.F.; Martins, I.S. Avaliação Da Concentração De Ferro Orgânico Em Uma População De Grávidas *. *Rev. Saúde públ.* **1983**.

63. Demétrio, F.; Teles-Santos, C.A. de S.; dos Santos, D.B. Insegurança Alimentar, Cuidado Pré-Natal e Outros Determinantes Da Anemia Em Mulheres Grávidas Da Coorte Nisami, Brasil: Modelo Conceitual Hierárquico. *Rev. Bras. Ginecol. e Obstet.* **2017**, *39*, 384–396, doi:10.1055/s-0037-1604093.

64. Lima, M. dos S. Deficiência De Vitamina D, Anemia Gestacional E Desfechos Perinatais: Um Estudo De Coorte, Universidade Estadual de Feira de Santana, 2021.

65. Silva, J.D.; Santos, D.B.; Silva, C.A.L.; Santos, M.P.; Araújo, E.M. DETERMINANTES DA ANEMIA EM UMA COORTE DE GESTANTES NO RECÔNCAVO BAIANO. **2020**.

66. Santos, E.M.F.; de Amorim, L.P.; Costa, O.L.N.; Oliveira, N.; Guimarães, A.C. Perfil de Risco Gestacional e Metabólico No Serviço de Pré-Natal de Maternidade Pública Do Nordeste Do Brasil. *Rev. Bras. Ginecol. e Obstet.* **2012**, *34*, 102–106.

67. Faria, D.G.S. de Perfil de Mães Adolescentes de São José Do Rio Preto e Cuidados Na Assistência Pré-Natal. *Fire extinguisher Perform. Eval. with GelTech Solut. inc.’s FireIce water Addit. Cl. 2-A 40-A cribs A ten-tire fire Gen. Accord. with UL 711* **2007**.

68. Anna Luiza Maffessoni, Naura Tonin Angonese, B.M.R. Perfil Epidemiológico Das Gestações Não Planejadas Em Um Hospital de Referência No Oeste Do Paraná. **2021**, *4*, 1–23.

69. Ferracioli, P.L.R.V. Intercorrências Na Gestação Em Um Município Do, Universidade Estadual De Maringá, 2017, Vol. 4.

70. Novaes, E.S.; Oliveira, R.R.; Melo, E.C.; Varela, P.L.R.; Mathias, T.A. de F. Perfil Obstétrico De Usuárias Do Sistema Único De Saúde Após Implantação Da Rede Mãe Paranaense. 2015.

71. Meier, P.R.; Nickerson, H.J.; Clinic, M.; Olson, K.A.; Berg, R.L.; Meyer, J.A. *Prevention of Iron Deficiency Anemia in Adolescent and Adult Pregnancies*; 2002; Vol. 1;.

72. Rodrigues, L.P.; Jorge, S.R.P.F. Deficiência de ferro na gestação, parto e puerpério TT - The iron deficiency in pregnancy, labor and puerperium. *Rev. Bras. Hematol. Hemoter.* **2010**, *32*, 53–56, doi:10.1590/S1516-84842010005000057.

73. Cruz, R.D. Avaliação Da Deficiência de Ferro Durante o Processo Gestacional e Sua Relação Com o Consumo Alimentar e a Suplementação Com Ferro Avaliação Da Deficiência de Ferro Durante o Processo Gestacional e Sua Relação Com o Consumo Alimentar e a Suplementação Com, Universidade de São Paulo, 2009.

74. Aragão, F.K.S.; Almeida, A.L.; Nunes, S.F.L. Prevalência e Fatores Associados à Anemia Em Gestantes Atendidas Em Uma Maternidade Pública No Município de Imperatriz, Maranhão. *JMPHC | J. Manag. Prim. Heal. Care | ISSN 2179-6750* **2013**, *4*, 190, https://doi:10.14295/jmphc.v4i3.187.

75. Arruda, I.K.G. de Prevalência de Anemia Em Gestantes de Baixa Renda: Algumas Variáveis Associadas e Sua Repercussão No Recém-Nascido, Master's Thesis, Universidade de Pernambuco - UFPE, Recife, Brazil,1990.

76. Frick, G.G.; Frizzo, M.N. Prevalência de Anemia e Seus Fatores Determinantes Em Gestantes de Município Do Noroeste Do Estado Do RS. *Rev. Context. Saúde* **2018**, *18*, 69, https://doi:10.21527/2176-7114.2018.34.69-76.

77. Fujimori, E. Gravidez Na Adolescência: Estado Nutricional Referente Ao Ferro, Ph.D Thesis, Universidade de São Paulo, Brazil, **1994**, p.87.

78. Fujimori, E..; Laurenti, D.; Núñes de Cassana, L.M.; Oliveira, I.M.V. de; Szarfarc, S.C. Anemia e Deficiência de Ferro Em Gestantes Adolescentes. *Rev. Nutr.* **2000**, *13*, 177–184, https://doi:10.1590/S1415-52732000000300004.

79. Guerra, E.M.; Barretto, O.C. de O.; Vaz, A.J.; Silveira, M.B. Prevalência de Anemia Em Gestantes de Primeira Consulta Em Centros de Saúde de Área Metropolitana, Brasil. *Rev. Saude Publica* **1990**, *24*, 380–386, https://doi:10.1590/S0034-89101990000500005.

80. Lucyk, J.M. Perfil Antropométrico, Consumo Alimentar e Concentração de Hemoglobina Em Gestantes Assistidas No Hospital Universitário de Brasília, Master's Thesis, Universidade de Brasília - UnB, Brasília, Brazil, 2006.

81. Magalhães, E.I.S.; Maia, D.S.; Pereira Netto, M.; Lamounier, J.A.; Rocha, D. da S. Prevalência de Anemia e Determinantes Da Concentração de Hemoglobina Em Gestantes. *Cad. Saúde Coletiva* **2018**, *26*, 384–390, https://doi:10.1590/1414-462x201800040085.

82. Marion, M. Prevalência de Anemia e Hipotireoidismo Em Gestantes Atendidas No Pré-Natal Do Hospital Universitário de Santa Maria - RS (HUSM), Master's Thesis, Universidade Federal de Santa Maria, Brazil, 2013.

83. Miranda, V.I.A.; Santos, I.S.; Silveira, M.F. da; Silveira, M.P.T.; Pizzol, T. da S.D.; Bertoldi, A.D. Validade Do Autorrelato de Anemia e Do Uso Terapêutico de Sais de Ferro Durante a Gestação: Coorte de Nascimentos de 2015 de Pelotas, Rio Grande Do Sul, Brasil. *Cadernos Saúde Públ.* **2018**, *34* e00125517.

84. Neme, L.C.L.H.; Brognoli, A.F.; Szarfarc, S.; Cristina, A.; Oliveira, L.; Renata, C.; Silva, D.M. Estado Nutricional, Consumo de Ferro e Vitamina C e Níveis Sanguíneos de Hemoglobina de Gestantes. *Cadernos Escola Saúde.* **2017**, *1*, 149–164.

85. Oliveira, A.C.M.; Barros, A.M.R.; Ferreira, R.C. Fatores de Associados à Anemia Em Gestantes Da Rede Pública de Saúde de Uma Capital Do Nordeste Do Brasil. *Rev. bras. ginecol. Obs.* **2015**, *37*, 505–511.

86. Arruda, I.K.G. de Deficiência de Ferro, de Folato e Anemia Em Gestantes Atendidas Do Instituto Materno-Infantil de Pernambuco Magnitude, Fatores de Risco e Algumas Implicações Nos Seus Conceptos. Master’s Thesis, Departamento de Nutrição, Universidade Federal de Pernambuco, Recife, Brazil, 1997.

87. Paiva, A.A.; Rondó, P.H. de C.; Pagliusi, R.A.; Latorre, M. do R.D. de O.; Cardoso, M.A.A.; Gondim, S.S.R. Relationship between the Iron Status of Pregnant Women and Their Newborns. *Rev. saúde pública* **2007**, *41*, 321–327.

88. Papa, A.C.E.; Furlan, J.P.; Pasquelle, M.; Guazzelli, C.A.F.; Figueiredo, M.S.; Camano, L.; Mattar, R. A Anemia Por Deficiência de Ferro Na Grávida Adolescente: Comparação Entre Métodos Laboratoriais. *Rev. Bras. Ginecol. e Obs.* **2003**, *25*, 731–738, https://doi:10.1590/S0100-72032003001000006.

89. Quintans, A.M. Anemia Em Gestantes: Avaliação Das Usuárias Das Unidades Básicas de Saúde Do Município de Cabedelo-Paraíba, Master’s Thesis, Universidade Federal da Paraíba, João Pessoa, Brazil, 2011.

90. Rocha, D. S.; Netto, M.P.; Priore, S.E.; Lima, N.M.M.; Rosado, L.E.F.P.L.; Franceschini, S.C.C. Estado Nutricional e Anemia Ferropriva Em Gestantes: Relação Com o Peso Da Criança Ao Nascer. *Rev. Nutr.* **2005**, *18*, 481–489, https://doi:10.1590/S1415-52732005000400004.

91. Sinisterra Rodriguez, O.; Szarfarc, S.C.; Benicio, M.H. d’Aquino Anemia e Desnutrição Maternas e Sua Relação Com o Peso Ao Nascer. *Rev. saúde pública* **1991**, *25*, pp. 193–197.

92. Sakamoto, T.M. Hemoglobinopatias e Anemias Em Gestantes No Hospital Universitário de Campo Grande - MS. Master’s Thesis, Campo Grande, Brazil, **2008**, pp. 1–100.

93. Santana, M. de S.; Costa Filho, A.A.; Lisboa, C.S.; Viana, A.S.; Santos, D.B. Influência Da Anemia Em Gestantes Sobre o Peso Ao Nascer: Um Estudo Da Coorte de NISAMI. *Rev. baiana saúde pública* **2019**, *43*, 581–598.

94. Santos, P.N.P. Prevalência de Anemia Nas Gestantes Atendidas Em Unidades de Saúde Da Família Em Feira de Santana, Bahia, Entre Outubro de 2005 e Março de 2006. Master’s Thesis, Universidade Estadual de Feira de Santana Departamento de Saúde, Programa de Pós-Graduação Em Saúde Coletiva, Feira de Santana, Brazil, **2006**.

95. Santos, A.C.B. Frequência de Consumo de Frutas, Hortaliças e Produtos Ultraprocessados e Estado Nutricional de Gestantes de Cruzeiro Do Sul, Acre. Master’s Thesis, Universidade de São Paulo, Brazil, **2016**.

96. Santos, A.M.C.; Azeredo, V.B.; Boaventura, G.T. Estado Nutricional de Gestantes Adolescentes de Um Serviço Público de Referência Para Assistência Pré-Natal de Alto Risco. *Nutr. Bras.* **2009**, *8,* 344.

97. Bezerra, A.S.; Cardoso, V.V.B.P.; Barbosa, V.S.A. Estado Nutricional, Anemia e Parasitoses Intestinais Em Gestantes de Um Município Do Curimataú Paraibano. *Rev. APS* **2018**, *21*, 15910. https://doi:10.34019/1809-8363.2018.v21.15910.

98. Santos, F.F.; Conceição, S.I.O.; Monteiro, S.G. Anemia Em Gestantes Atendidas Em Maternidades, Em São Luís (MA). *Cad. Pesqui.* **2012**, *19*, 54–61.

99. Silva, D.F.S. Anemia Ferropriva e Fatores Associados Em Gestantes Assistidas Em Hospital de Referência Do Estado de Pernambuco. Master’s Thesis, Universidade Federal de Pernambuco, Recife, Brazil, 2012.

100. Silva, S.A.M. da S. Anemia Em Gestantes Usuárias Do Serviço Público de Saúde Em Santo Antônio de Jesus - BA. Master’s Thesis Universidade Federal do Recôncavo da Bahia, Cruz das Almas, Bahia, Brazil, 2014.

101. Silva, R.M. Determinantes Da Anemia Em Gestantes Usuárias Do Programa de Saúde Da Família No Município de Santo Antônio de Jesus - Bahia. Master’s Thesis, Universidad Estatal de Feira de Santana, Feira de Santana, Brazil, **2015**.

102. Soares, N.N.; Mattar, R.; Camano, L.; Torloni, M.R. Iron Deficiency Anemia and Iron Stores in Adult and Adolescent Women in Pregnancy. *Acta Obstet. Gynecol. Scand.* **2010**, *89*, 343–349, doi:10.3109/00016340903559992.

103. Soares, F.M.M.; Nunes, R. da S.; Henrique, I. da S.N.; Simão, A.L.S. Incidência de Anemia Ferropriva Em Gestantes Em Um Município de Pequeno Porte. **2021**, 15, 74–83.

104. Szarfarc, S.C. Anemia Ferropriva Em Parturientes e Recém-Nascidos. *Rev. Saude Publica* **1974**, *8*, 369–374, https://doi:10.1590/s0034-89101974000400003.

105. Camargo, R.; Pereira, R. Fatores Associados à Deficiência de Ferro Em Gestantes Atendidas Em Serviço Público de Pré-Natal. *Rev. Nutr.* **2013**, *26*, 455–464.

106. Côrtes, M.H. Impacto Da Fortificação Das Farinhas de Trigo e de Milho Com Ferro Nos Níveis de Hemoglobina Das Gestantes Atendidas Pelo Pré-Natal Do Hospital Universitário de Brasília/DF. Master's Thesis, Universidade de Brasília, Brazil, 2006.

107. Costa, C.M.; Brum, I.R.; Lima, E.S. Anemia e Marcadores Séricos Da Deficiência de Ferro Em Grávidas Atendidas Na Rede Pública Municipal de Manaus, Amazonas, Brasil. *Acta Amaz.* **2009**, *39*, 901–905, https://doi:10.1590/S0044-59672009000400018.

108. Dias, A.C.P. Anemia Ferropriva Em Gestantes Adolescentes Atendidas Em Postos de Saúde de Araraquara-SP. Master's Thesis, Universidade Estadual Paulista Júlio de Mesquita Filho, Araraquara, Brazil, 2000.

109. Ferreira, H.S.; Moura, F.A.; Cabral Júnior, C.R. Prevalência e Fatores Associados à Anemia Em Gestantes Da Região Semi-Árida Do Estado de Alagoas. *Rev. Bras. Ginecol. e Obs.* **2008**, *30*, 445–451, https://doi:10.1590/S0100-72032008000900004.

110. Figueiredo ACMG, G.-F.I.; Batista JET, Orrico GS, Porto ECL, C.P.; RM, et al. Maternal Anemia and Birth Weight: A Prospective Cohort Study. *PLoS One* **2019**, *14*, e0212817, https://doi:10.1371/journal.pone.0212817.
